# Surveillance, Detection and Therapeutic Innovation in Antimicrobial Resistance in the Age of Polycrisis: From Genomic and Digital Innovation to One Health Integration

**DOI:** 10.3390/microorganisms14102308

**Published:** 2026-10-10

**Authors:** Francesco Branda, Francesco De Maria, Ivailo Alexiev, Ayşe Banu Demir, Önder Limon, Giancarlo Ceccarelli, Fabio Scarpa, Alessandro Russo, Silvia Spoto, Massimo Ciccozzi

**Affiliations:** 1 Unit of Medical Statistics and Molecular Epidemiology, Università Campus Bio-Medico di Roma, 00128 Rome, Italy; m.ciccozzi@unicampus.it; 2 Infectious and Tropical Diseases Unit, “Renato Dulbecco” Teaching Hospital of Catanzaro, 88100 Catanzaro, Italy; francescodemaria16@gmail.com (F.D.M.); a.russo@unicz.it (A.R.); 3 National Reference Confirmatory Laboratory of HIV, National Center of Infectious and Parasitic Diseases (NCIPD), 1504 Sofia, Bulgaria; ivoalexiev@ncipd.org; 4 Department of Medical Biology, Faculty of Medicine, İzmir University of Economics, İzmir 35330, Türkiye; banu.demir@izmirekonomi.edu.tr; 5 Faculty of Medicine, IUE Medicalpoint Hospital, Yeni Girne Av 1825 S No:12, Karşıyaka, İzmir 35575, Türkiye; onder.limon@izmirekonomi.edu.tr; 6 Department of Public Health and Infectious Diseases, University Hospital Policlinico Umberto I, Sapienza University of Rome, 00185 Rome, Italy; giancarlo.ceccarelli@uniroma1.it; 7 Department of Biomedical Sciences, University of Sassari, 07100 Sassari, Italy; fscarpa@uniss.it; 8 Diagnostic and Therapeutic Medicine Department, Fondazione Policlinico Universitario Campus Bio-Medico, Via Alvaro del Portillo, 200, 00128 Rome, Italy

**Keywords:** antimicrobial resistance, genomic surveillance, wastewater surveillance, One Health, artificial intelligence, machine learning, whole-genome sequencing, metagenomics, digital surveillance, health inequalities

## Abstract

Antimicrobial resistance (AMR) surveillance is increasingly strained by interconnected global crises, i.e., pandemics, climate change, armed conflicts, population mobility, and health inequalities, that disrupt healthcare delivery, diagnostic capacity, and laboratory infrastructure. In parallel, artificial intelligence (AI), machine learning, whole-genome sequencing, metagenomics, and digital surveillance offer the potential to shift from reactive reporting to predictive intelligence that anticipates resistance threats, although current implementations remain sector-specific, retrospective, and project-funded. Environmental surveillance, particularly wastewater monitoring coupled with genomic epidemiology, provides early warnings at population and ecosystem levels. The future integration of AI across human, animal, and environmental sectors could enable unified One Health intelligence; no such integrated platform is currently operational. This review focuses primarily on bacterial AMR. It examines how global drivers strain surveillance, how genomic, metagenomic, digital, and AI-enabled technologies reshape AMR detection and prediction, and the emerging non-antibiotic therapeutic landscape, including bacteriophage therapy, Clustered Regularly Interspaced Short Palindromic Repeats (CRISPR)-based antimicrobials, antimicrobial peptides, microbiota-directed interventions, nanomedicine, host-directed therapies, photodynamic therapy, and vaccines. It then explores how these innovations can be integrated within a One Health framework to support coordinated global preparedness. Addressing the AMR crisis will require not only technological innovation but also equitable implementation, interdisciplinary collaboration, and resilient systems.

## 1. Introduction

Antimicrobial resistance (AMR) has emerged as one of the greatest threats to global health, undermining decades of progress in the treatment and prevention of infectious diseases [1]. Once viewed primarily as a microbiological consequence of inappropriate antibiotic use, AMR is now understood as a complex, dynamic phenomenon shaped by biological, environmental, social, economic, and political determinants [2]. The growing prevalence of multidrug-resistant (MDR), extensively drug-resistant (XDR), and pan-drug-resistant (PDR) pathogens is progressively diminishing the effectiveness of available antimicrobial therapies, leading to increased morbidity, mortality, and healthcare costs, while heightening the risk of treatment failure across virtually every field of modern medicine [3,4].

The global burden of AMR is staggering. According to the Global Burden of Disease study, bacterial AMR was directly responsible for approximately 1.27 million deaths in 2019 and contributed to nearly five million deaths overall, making it one of the leading causes of mortality worldwide [5]. Updated estimates place the number of deaths associated with bacterial AMR at 4.71 million in 2021 and project that resistant infections could contribute to as many as 39 million deaths between 2025 and 2050 if current trends persist [6]. These numbers likely underestimate the true impact, as resistance rates continue to rise in both community and healthcare settings despite ongoing antimicrobial stewardship programs. Recent data from the WHO Global Antimicrobial Resistance and Use Surveillance System (GLASS) confirm the sustained global expansion of resistant pathogens, underscoring that AMR has become a persistent public health emergency rather than a future threat [7].

Crucially, the epidemiology of AMR can no longer be explained by antibiotic consumption alone. While inappropriate prescribing and overuse remain key drivers, a growing body of evidence points to a confluence of interconnected global pressures that are accelerating resistance [4,8]. The COVID-19 pandemic, for instance, severely disrupted antimicrobial stewardship and healthcare delivery, resulting in a surge of empirical antibiotic use and delayed microbiological diagnosis, which in turn accelerated the selection of resistant organisms [9,10,11]. Concurrently, climate change is reshaping environmental reservoirs of resistance genes through rising temperatures, extreme weather events, and altered ecological interactions that promote bacterial dissemination [12,13]. Surface waters are increasingly recognised as a distinct compartment in this process: genomic analyses of *Klebsiella pneumoniae* recovered from rivers, lakes and treated effluents worldwide have shown that environmental isolates frequently carry the same resistance and virulence determinants found in clinical strains, indicating that aquatic environments act not merely as passive sinks but as reservoirs with direct clinical relevance [14]. Armed conflicts have devastated healthcare infrastructure, weakened infection prevention and control measures, and promoted uncontrolled antibiotic use, thereby facilitating the emergence and cross-border spread of antimicrobial-resistant pathogens [15,16,17,18,19]. At the same time, population displacement and increasing global mobility accelerate the international spread of resistant bacteria [20,21,22], while persistent health inequalities continue to restrict access to diagnostics and evidence-based therapies in many low- and middle-income countries [23,24].

Together, these overlapping crises have transformed AMR into a paradigmatic example of a global polycrisis [25], in which interconnected systemic challenges reinforce one another, amplifying their impacts across health, environmental, economic, and social systems. Healthcare disruption, environmental degradation, geopolitical instability, urbanization, food production systems, and socioeconomic disparities collectively create conditions that favor the emergence and dissemination of resistance. Consequently, AMR should no longer be viewed solely as an isolated biomedical problem, but as a systemic consequence of an increasingly interconnected world [4,26].

Addressing this complexity demands a fundamental shift in strategy. While antimicrobial stewardship and antibiotic development remain essential, they are no longer sufficient. The next generation of AMR control will depend on integrating a suite of technological innovations capable of enhancing surveillance, accelerating diagnostics, predicting resistance patterns, optimizing therapy, and discovering novel compounds.

Artificial intelligence (AI) and machine learning are already playing a pivotal role in predicting resistance patterns and supporting clinical decision-making [27,28,29]. These tools can integrate vast datasets, including microbiological findings, clinical variables, and antimicrobial consumption, to generate actionable insights that were previously unattainable [30,31]. Meanwhile, whole-genome sequencing and metagenomics are revolutionizing our ability to characterize resistance determinants, track transmission dynamics, and identify pathogens directly from clinical specimens without the need for prior culture [32,33,34]. Digital epidemiology and predictive modeling further enhance these capabilities by enabling real-time surveillance and forecasting of resistance trends across populations and geographical regions [35,36,37]. Collectively, these technologies are already reshaping how AMR is detected, monitored, and managed, offering capabilities that were unimaginable a decade ago.

At the same time, novel therapeutic paradigms are emerging. Advances in bacteriophage therapy, Clustered Regularly Interspaced Short Palindromic Repeats (CRISPR)-based antimicrobials, antimicrobial peptides, microbiome modulation, nanomedicine, and host-directed immunotherapies suggest that the future management of resistant infections will extend well beyond conventional antibiotics [2,3]. Rather than replacing existing agents, these complementary strategies aim to diversify treatment options while reducing selective pressure for resistance.

The convergence of these technological advances with the One Health framework offers a significant opportunity to rethink the global response to AMR. By integrating human, animal, and environmental surveillance with AI, genomic epidemiology, and coordinated international policies, it may become possible to transition from a reactive model, focused on treating infections after they occur, to a proactive system capable of predicting, preventing, and mitigating resistance before it spreads widely [32,38,39,40,41,42,43].

**Figure 1:**
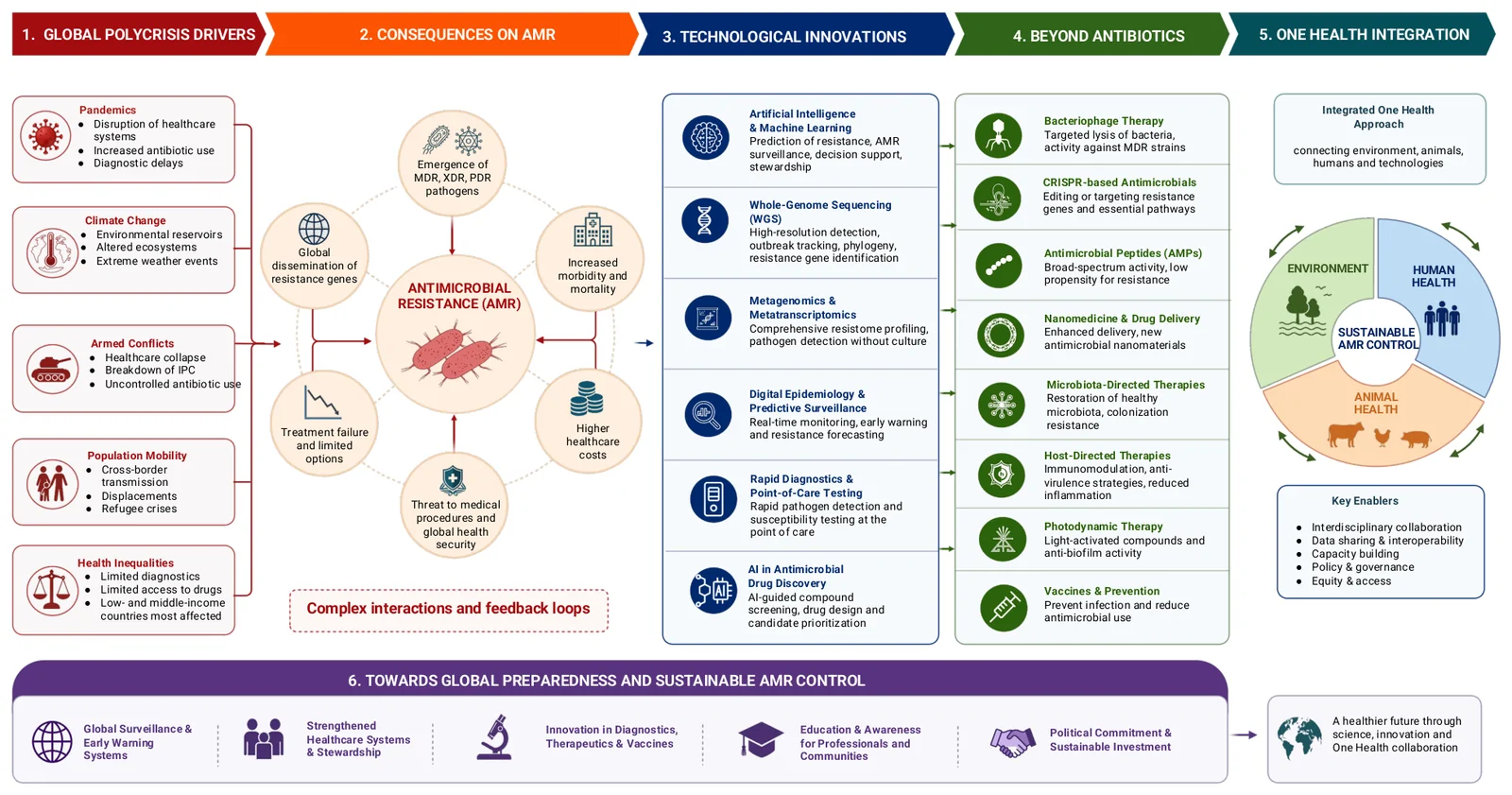
**Conceptual framework illustrating how multiple interconnected global drivers, including pandemics, climate change, armed conflicts, population mobility, and health inequalities, collectively accelerate the emergence and dissemination of AMR.** The figure highlights the transition from traditional antimicrobial stewardship toward an integrated strategy based on technological innovation, including artificial intelligence, machine learning, genomics, metagenomics, digital surveillance, and predictive modelling, together with emerging non-antibiotic therapeutic approaches such as bacteriophage therapy, CRISPR-based antimicrobials, antimicrobial peptides, microbiota-directed interventions, nanomedicine, host-directed therapies, and photodynamic therapy. These complementary advances converge within the One Health framework, integrating human, animal, and environmental health to support predictive surveillance, coordinated prevention, and sustainable global preparedness against AMR. Abbreviations: AMR, antimicrobial resistance; AI, artificial intelligence; ML, machine learning; WGS, whole-genome sequencing; MDR, multidrug-resistant; XDR, extensively drug-resistant; PDR, pan-drug-resistant; CRISPR, clustered regularly interspaced short palindromic repeats.

In this narrative review, we examine the major forces accelerating AMR in the context of today’s global polycrisis and discuss how emerging technologies and innovative therapeutic strategies are redefining the future of prevention and control. We also explore how these advances can be integrated within a comprehensive One Health framework to strengthen global preparedness against one of the defining public health challenges of the twenty-first century (Figure 1).

Several recent reviews have addressed adjacent territory, and it is useful to state at the outset how the present work differs from them. Jangid et al. examine One Health strategies against AMR through the combined lens of artificial intelligence, genomics and environmental surveillance, framed around planetary health [44]. Kasse et al. provide a scoping review of the opportunities and challenges of applying AI within One Health approaches to AMR, with an emphasis on mapping the evidence base and its methodological quality [45]. Salama et al. offer a narrative synthesis of AI applications across the AMR response [46], while Lawal et al. focus specifically on AI-integrated biosensors for AMR detection and surveillance and their implications for global biosecurity [47]. Each of these contributions treats a technological or ecological dimension of the problem in depth, and we draw on all four in the sections that follow.

What distinguishes the present review is not the addition of a further technology-centred synthesis but the attempt to hold two bodies of literature in a single narrative arc. The first concerns the structural drivers that are currently degrading surveillance capacity, for example the aftermath of the COVID-19 pandemic, climate change, armed conflict, population mobility and health inequalities, which are typically analysed within the political-epidemiology literature and are largely absent from technology-focused reviews. The second concerns the genomic, digital and AI-enabled response, together with the emerging non-antibiotic therapeutic landscape. Our argument is that these cannot be assessed independently: the value of a predictive surveillance architecture is determined by the conditions of the systems in which it must operate, and those conditions are precisely what the polycrisis is eroding. We therefore organise the review as driver, response and integration, rather than as a catalogue of technologies, and we treat the One Health framework not as a concluding aspiration but as the operational architecture in which the mismatch between the two bodies of literature has to be resolved.

To ensure transparency and reproducibility of the literature selection, we conducted a structured but non-systematic literature search. PubMed/MEDLINE, Scopus, and Web of Science were searched from January 2015 to June 2026 using combinations of the terms “antimicrobial resistance”, “antibiotic resistance”, “One Health”, “artificial intelligence”, “machine learning”, “whole-genome sequencing”, “metagenomics”, “wastewater surveillance”, “bacteriophage therapy”, “CRISPR”, “antimicrobial peptides”, “nanomedicine”, and “host-directed therapy”. Only English-language publications were considered. The search was restricted to bacterial AMR. Antifungal and antiviral resistance are adjacent and clinically significant challenges that share some of the same drivers and surveillance technologies, but they require dedicated reviews and are not systematically covered here. Priority was given to systematic reviews, meta-analyses, consensus statements, and documents issued by WHO and the Quadripartite organisations, supplemented by primary studies where these provided the only available evidence.

The search retrieved a large volume of records from the three databases. After removal of duplicates, records were screened by title and abstract; those not relevant to the scope of the review, not reporting primary or synthesised evidence, or addressing resistance in non-bacterial pathogens only were excluded. The remaining records were assessed in full text, and further exclusions were applied for the same reasons together with the absence of extractable methodological detail. Additional documents were identified through manual screening of the reference lists of retrieved articles and through targeted searching of WHO and Quadripartite repositories. The final set of documents informing this review is reflected in the reference list.

Studies were included if they: (i) addressed bacterial AMR surveillance, detection, or therapeutic strategies; (ii) were published in English between January 2015 and June 2026; and (iii) reported primary data, systematic reviews, meta-analyses, consensus statements, or authoritative guidance from WHO and the Quadripartite organisations. Studies were excluded if they: (i) addressed only antifungal or antiviral resistance without relevance to bacterial AMR; (ii) were editorials, commentaries, or opinion pieces without supporting data; (iii) reported only in vitro or animal data without translational relevance to human surveillance or therapy; or (iv) were conference abstracts or preprints without peer review.

Because this is a narrative review rather than a systematic one, study quality was not assessed through a formal risk-of-bias tool or quantitative scoring system. Instead, the following hierarchy of evidence was applied during selection: (i) systematic reviews and meta-analyses were prioritised for established topics; (ii) randomised controlled trials and prospective clinical studies were prioritised for therapeutic and diagnostic claims; (iii) WHO and Quadripartite documents were prioritised for governance and policy statements; and (iv) primary studies were cited only where they provided the only available evidence or where they exemplified a specific technology or implementation. Where evidence was heterogeneous or only preclinical, this is stated explicitly in the relevant section.

As this is a narrative review, the search was structured but not systematic: the terms, databases, and date limits were pre-specified, but no formal protocol was registered, no PRISMA-compliant screening log was maintained, and neither a risk-of-bias assessment nor a quantitative synthesis was performed. Selection within the eligible set was purposive rather than exhaustive, and the description above is not intended to imply a systematic search. Exact screening counts were not retained during the initial selection process and could not be reconstructed accurately post hoc without re-running the searches with the original date limits.

The work is structured into seven main sections. Section 2 examines the principal drivers accelerating AMR, including the COVID-19 pandemic, climate change, armed conflicts, migration, mobility, and health inequalities. Section 3 explores technological innovations in AMR control, covering artificial intelligence, genomics, metagenomics, digital surveillance, rapid diagnostics, and drug discovery. Section 4 reviews emerging non-antibiotic therapeutic strategies, including bacteriophage therapy, CRISPR-based antimicrobials, antimicrobial peptides, nanomedicine, microbiota-directed therapies, host-directed immunotherapy, and photodynamic therapy. Section 5 presents the One Health framework, emphasizing the interconnection between human, animal, and environmental health and the role of AI as a connective infrastructure. Section 6 provides a critical discussion of current limitations, the challenges of technological innovation, global inequities, and governance needs, integrating a synthesis of priority areas for coordinated action. Finally, Section 7 offers concluding remarks and outlines priorities for future research and policy action.

## 2. Why Surveillance Systems Are Under Strain: Drivers of Accelerating AMR and Their Implications for Detection

Effective AMR surveillance depends on robust laboratory infrastructure, timely microbiological diagnosis, reliable data reporting, and coordinated information sharing across sectors. However, each of the global crises discussed below has directly undermined these foundations, disrupting surveillance workflows, compromising data quality, and creating gaps that allow resistance to spread undetected. Understanding these pressures is essential for designing resilient surveillance systems capable of withstanding future shocks.

### 2.1. The Legacy of the COVID-19 Pandemic

The COVID-19 pandemic marked a turning point in the epidemiology of antimicrobial resistance, altering the trajectory of resistance emergence across healthcare systems worldwide. During the initial phases of the pandemic, uncertainty regarding bacterial co-infections, overwhelmed healthcare infrastructure, and limited diagnostic capacity collectively resulted in a marked surge in empirical antibiotic prescribing [9]. Although confirmed bacterial infections were relatively uncommon among hospitalized COVID-19 patients, antibiotics were administered to the great majority of these individuals, frequently before any microbiological confirmation was obtained; the pooled discrepancy between the prevalence of confirmed bacterial co-infection and the proportion of patients actually receiving antibacterial therapy is one of the most consistent findings of the pandemic literature [10,11].

This widespread and often unnecessary use of broad-spectrum antibiotics occurred simultaneously with major disruptions in established antimicrobial stewardship programmes. Infection prevention and control activities were rapidly redirected toward pandemic containment, microbiological laboratories experienced substantial operational challenges including staff shortages and supply chain interruptions, and routine surveillance systems were frequently interrupted or entirely suspended [9,10]. Prolonged intensive care unit admissions, increased utilization of invasive devices such as mechanical ventilators and central venous catheters, and persistent overcrowding in hospital settings further created an ideal environment for healthcare-associated transmission of multidrug-resistant organisms [9].

Recent systematic reviews and meta-analyses consistently demonstrate that the COVID-19 pandemic was associated with significantly increased rates of infections caused by methicillin-resistant *Staphylococcus aureus* (MRSA), carbapenem-resistant *Acinetobacter baumannii*, multidrug-resistant *Pseudomonas aeruginosa*, and carbapenem-resistant Enterobacterales, particularly in intensive care settings [9,10,11]. Importantly, these concerning effects have persisted well beyond the acute phases of the pandemic, suggesting that COVID-19 acted not merely as a temporary disruption but as a powerful accelerator of pre-existing resistance trends that will continue to affect patient outcomes for years to come [11].

Perhaps the most important lesson emerging from the COVID-19 experience is that antimicrobial stewardship cannot be considered an isolated clinical intervention. Future pandemic preparedness plans must integrate stewardship principles, rapid diagnostic capabilities, genomic surveillance infrastructure, and robust infection prevention measures from the earliest stages of emerging infectious disease responses. Without such integration, every major epidemic risks generating a secondary, and potentially more devastating, pandemic of antimicrobial resistance.

### 2.2. Climate Change and Environmental Pressures

Environmental change has emerged as another major determinant of antimicrobial resistance, operating at scales that transcend traditional healthcare boundaries. Traditionally viewed as a problem largely confined to hospitals and clinical settings, AMR is now increasingly recognised as an ecological phenomenon involving complex, bidirectional interactions among humans, animals, water systems, soil, wildlife, and agricultural practices [12]. Climate change substantially amplifies these interactions by fundamentally altering the environmental conditions that favour bacterial survival, horizontal gene exchange, and pathogen dissemination across ecosystems [13,40]. The most conspicuous climate-linked example of an emerging resistant pathogen is not bacterial but fungal: *Candida auris*, a multidrug-resistant yeast first isolated in 2009, has since spread globally and is now recognised by WHO as a critical-priority fungal pathogen. Although the causal link between climate change and its emergence remains debated, several hypotheses invoke thermotolerance and ecosystem disruption as contributing factors [48]. This example reinforces the argument that AMR surveillance must be designed to detect emerging resistance across pathogen kingdoms, not only within bacteria.

Rising global temperatures have been associated, largely in ecological and modelling studies, with increased bacterial growth rates and enhanced horizontal gene transfer, potentially facilitating the spread of resistance determinants across bacterial populations in both clinical and environmental settings [12]. Extreme weather events, including floods, droughts, hurricanes, and prolonged heatwaves, can disrupt sanitation infrastructure, contaminate freshwater sources with antimicrobial residues and resistant bacteria, and disseminate resistant microorganisms over wide geographical areas through water runoff and aerosolization. At the same time, changing rainfall patterns significantly influence agricultural runoff, increasing the environmental circulation of both antibiotics and resistance genes from farming operations into natural water systems [12].

Wastewater treatment plants, surface waters, aquaculture systems, and agricultural environments have become increasingly recognised as critical reservoirs of the environmental resistome, where resistant bacteria originating from human, veterinary, and industrial sources coexist and actively exchange genetic material through conjugation, transformation, and transduction [13,38]. These interconnected ecosystems provide ideal conditions for the emergence of novel resistance mechanisms and their subsequent introduction into clinical settings, creating a continuous cycle of resistance gene exchange between environmental and healthcare reservoirs [40,41].

The environmental dimension of AMR is therefore no longer peripheral or secondary but represents one of its fundamental and most challenging components. Effective surveillance strategies must increasingly include comprehensive environmental monitoring, including wastewater surveillance, agricultural runoff analysis, and wildlife screening, alongside traditional hospital- and community-based surveillance programmes [49,50,51,52]. It should be acknowledged, however, that much of the evidence linking climate variables to clinical resistance is ecological and correlational; causal pathways remain difficult to isolate, and this uncertainty should temper the strength of inference drawn from such studies. This integrated approach nonetheless reinforces the rationale for a comprehensive One Health framework that recognizes the inseparability of human, animal, and environmental health in addressing antimicrobial resistance [41].

This is not a theoretical concern. Comparative genomic work on *Klebsiella pneumoniae* isolated from surface waters across several continents has documented environmental populations carrying carbapenemase and extended-spectrum β-lactamase genes, together with virulence determinants associated with hypervirulent clinical lineages, and has shown phylogenetic proximity between aquatic and clinical isolates [14]. Surface waters therefore represent a compartment in which clinically consequential genotypes both persist and circulate, which is the empirical basis for the surveillance arguments developed in Section 3.4.

### 2.3. Armed Conflicts and Humanitarian Crises

War represents one of the most powerful yet consistently underappreciated accelerators of antimicrobial resistance, creating conditions that are uniquely conducive to the emergence, amplification, and international spread of resistant pathogens. Armed conflicts profoundly disrupt healthcare delivery systems, damage laboratory infrastructure, interrupt established antimicrobial stewardship programmes, and severely limit access to diagnostics, infection prevention measures, and appropriate antimicrobial therapy [15]. Under these extreme circumstances, empirical broad-spectrum antibiotic use frequently becomes the only clinically feasible option, inevitably driving resistance selection in vulnerable patient populations [18].

Recent conflicts, including those in Ukraine and Gaza, have starkly highlighted how modern warfare creates ideal conditions for the emergence and dissemination of resistant pathogens. Hospitals operating under extreme resource constraints often face critical shortages of microbiological testing capabilities, inadequate sterilisation and disinfection procedures, chronic overcrowding, and severely reduced infection control capacity [17]. Simultaneously, traumatic injuries from explosive devices and projectiles, complex surgical procedures, extensive burns, and prolonged hospitalisations substantially increase the risk of healthcare-associated infections caused by multidrug-resistant bacteria, further compounding the burden on already overwhelmed healthcare systems [15,17,19].

Conflict also facilitates the international spread of resistance through the large-scale displacement of patients, healthcare workers, and civilian populations across national borders [17]. Patients evacuated from active conflict zones have repeatedly been reported to carry carbapenemase-producing Enterobacterales and *Acinetobacter baumannii* with unusually broad resistance profiles, prompting dedicated admission-screening recommendations in receiving countries [15,17,18]. Individuals requiring specialised medical care are frequently transferred between countries, creating additional opportunities for resistant organisms to disseminate between healthcare systems with different resistance epidemiology and infection control practices. The consequences of conflict-driven AMR therefore extend well beyond immediate conflict zones, reinforcing the concept that antimicrobial resistance is a shared global security challenge rather than a local clinical problem confined to war-affected regions [16].

Increasingly, AMR is being recognised as one of the “invisible casualties” of armed conflict. Unlike immediate traumatic injuries, the effects of conflict-driven antimicrobial resistance may continue for years or even decades after hostilities cease, profoundly influencing healthcare systems both within and beyond affected regions. This long-lasting impact underscores the urgent need for comprehensive AMR preparedness in conflict settings and humanitarian responses [16].

### 2.4. Migration, Mobility, and Health Inequalities

Human mobility has always influenced the epidemiology of infectious diseases, but contemporary globalisation has greatly accelerated the international movement of resistant microorganisms across continents and regions. International travel, labour migration, medical tourism, forced displacement due to conflict or environmental disasters, and refugee movements all contribute to the global circulation of antimicrobial resistance, often bridging regions with markedly different resistance epidemiology, healthcare practices, and antimicrobial availability [22].

Importantly, migrants and refugees should not be viewed as drivers of resistance themselves but rather as populations disproportionately exposed to conditions that facilitate acquisition, carriage, and transmission of resistant organisms. Overcrowded living environments, disrupted healthcare access, limited sanitation infrastructure, and interrupted antimicrobial treatment all substantially increase the likelihood of colonisation by multidrug-resistant organisms [20]. Systematic reviews consistently indicate higher rates of carriage of resistant Enterobacterales, MRSA, and other MDR pathogens among displaced populations, particularly following prolonged stays in refugee camps or conflict-affected settings where infection prevention and control measures are often severely compromised [21].

At the same time, increasing international connectivity through air travel and global commerce allows resistant strains to spread rapidly between continents through asymptomatic colonisation of healthy individuals. This phenomenon underscores the critical importance of coordinated surveillance systems capable of integrating microbiological data across national borders and sharing epidemiological intelligence in real-time. AMR surveillance can no longer remain confined within individual healthcare systems or national boundaries but must increasingly reflect the realities of an interconnected globalised world where pathogens and resistance genes move freely [22].

Perhaps the most pervasive and intractable driver of antimicrobial resistance is the inequitable distribution of healthcare resources and diagnostic capacity [42]. In many low- and middle-income countries, limited laboratory infrastructure, severe shortages of trained personnel, restricted access to microbiological diagnostics, and inconsistent antimicrobial availability compel clinicians to rely on empirical treatment strategies [23]. Although often clinically unavoidable, this approach substantially increases unnecessary antibiotic exposure and strongly favours the selection of resistant pathogens, perpetuating a vicious cycle of resistance emergence in resource-limited settings [24].

The diagnostic gap represents one of the greatest barriers to effective antimicrobial stewardship globally. Without timely identification of the causative pathogen and its susceptibility profile, clinicians have no alternative but to prescribe broad-spectrum antibiotics to minimise the immediate risk of treatment failure [23]. Yet it is equally important to recognise that lack of access to effective antibiotics still causes more deaths than resistance itself in many low-income settings; the 2024 United Nations Political Declaration therefore pairs stewardship commitments with an explicit access target, namely that at least 70% of human antibiotic consumption should come from the WHO Access group of the AWaRe classification [53,54]. While this pragmatic strategy may benefit individual patients in resource-limited settings, its cumulative population-level effect contributes powerfully to the progressive expansion of antimicrobial resistance, particularly affecting vulnerable populations already marginalised by weak healthcare systems [43].

Socioeconomic inequalities further amplify this vicious cycle through limited access to clean water and basic sanitation, inadequate vaccination programmes, absent infection prevention measures, and unaffordable essential medicines [24]. Consequently, AMR disproportionately affects regions that are least equipped to detect, respond to, and treat resistant infections, creating a self-reinforcing cycle in which weak health systems promote resistance, while resistance further weakens already fragile healthcare infrastructures and undermines economic development [43].

Addressing antimicrobial resistance therefore requires far more than scientific innovation alone. Strengthening laboratory networks, expanding access to rapid and affordable diagnostics, investing in healthcare infrastructure and workforce training, and reducing global socioeconomic inequities are indispensable components of any sustainable and equitable AMR strategy. Without these foundational investments, technological advances will remain inaccessible to those who need them most.

Although each of these factors contributes independently to antimicrobial resistance, their most devastating impact arises from their complex interactions and synergistic amplification. Climate-related disasters often precipitate humanitarian crises and population displacement; armed conflicts drive forced migration and destroy healthcare infrastructure; fragile healthcare systems are disproportionately affected by pandemics and climate events; and diagnostic limitations amplify inappropriate antibiotic use across all these contexts simultaneously [8]. The result is a self-reinforcing network of biological, environmental, and societal processes that collectively accelerate the emergence and global spread of resistant pathogens far beyond what any single driver could achieve independently.

This profound interconnectedness explains why antimicrobial resistance should no longer be interpreted solely through the narrow lens of clinical microbiology. Instead, AMR represents the biological consequence of an increasingly unstable and interconnected global system, where ecological disruption, geopolitical instability, technological disparities, and healthcare inequalities converge to fundamentally reshape the epidemiology of infectious diseases [4,25,26]. Recognising this broader systemic context is essential for designing effective and sustainable responses, as the solutions to AMR must be as multidimensional and interconnected as the forces driving it. Addressing antimicrobial resistance in the twenty-first century requires not only scientific and technological innovation but also political commitment, international cooperation, and a fundamental commitment to health equity and social justice.

Taken together, the drivers described in this section share a common effect on detection capacity rather than only on resistance itself. Each of them removes, degrades or delays the microbiological information on which surveillance depends: the pandemic through the suspension of routine reporting, climate-related events through the disruption of sanitation and sampling infrastructure, conflict through the destruction of laboratories, mobility through the fragmentation of patient records across jurisdictions, and inequality through the absence of diagnostic capacity in the first place. The technologies reviewed in Section 3 must therefore be judged not only by their analytical performance but by their resilience under precisely these conditions. To avoid repetition, the environmental, climatic and mobility dimensions are treated substantively here and are referred to, rather than re-described, in the sections that follow.

## 3. Surveillance and Diagnostic Technologies for AMR

The growing complexity of antimicrobial resistance demands solutions that extend well beyond traditional microbiological approaches. While antimicrobial stewardship remains the cornerstone of resistance control, the unprecedented volume of microbiological, genomic, epidemiological, and clinical data generated by modern healthcare systems has exceeded the capacity of conventional analytical methods [4]. Advances in artificial intelligence (AI), machine learning (ML), high-throughput sequencing technologies, and digital epidemiology are fundamentally transforming the way resistant pathogens are identified, monitored, and managed [7,8]. Rather than representing isolated innovations, these technologies increasingly function as complementary components of an integrated ecosystem capable of supporting precision diagnostics, real-time surveillance, predictive modelling, and accelerated antimicrobial discovery [27,32,35].

Unlike conventional surveillance systems, which largely rely on retrospective analysis of laboratory-confirmed cases, emerging digital technologies offer the unprecedented possibility of anticipating resistance trends before they become clinically apparent [28,29]. This fundamental transition from reactive to predictive medicine may represent one of the most important paradigm shifts in the history of infectious diseases, potentially enabling pre-emptive interventions that could significantly reduce the burden of resistant infections [30,37].

### 3.1. AI and ML for Clinical Decision Support

AI has rapidly evolved from an experimental research tool into a clinically relevant technology with applications spanning virtually every stage of antimicrobial resistance management. By identifying complex, non-linear patterns within multidimensional datasets, AI algorithms can integrate diverse information sources, including microbiological findings, clinical variables, imaging data, laboratory results, antimicrobial consumption patterns, and genomic information, to generate predictions that would be difficult or impossible to achieve using traditional statistical approaches [28,29].

Among the various AI methodologies, machine learning has attracted particular attention because of its ability to continuously improve predictive performance as additional data become available through iterative training and validation [37]. Supervised learning models can accurately classify bacterial susceptibility profiles, estimate the probability of resistance to specific antimicrobial agents, and support empirical antibiotic selection before conventional susceptibility testing becomes available [30]. More advanced deep learning architectures, including convolutional neural networks and transformer-based models, are increasingly capable of analysing highly complex datasets, including whole-genome sequences and metagenomic data, thereby expanding the potential applications of AI within clinical microbiology [27,28,31].

These technologies are particularly valuable in time-critical clinical scenarios such as sepsis, bloodstream infections, and severe pneumonia, where early optimisation of antimicrobial therapy is directly associated with improved patient survival and reduced morbidity [29]. By rapidly estimating the likelihood of resistance based on patient characteristics, local epidemiological data, and prior antimicrobial exposure, AI-assisted decision support systems may facilitate earlier administration of appropriate therapy while simultaneously reducing unnecessary exposure to broad-spectrum antibiotics that drive resistance selection [30,35].

Nevertheless, current AI models remain heavily dependent on the quality, completeness, and representativeness of the data used for training. Algorithms developed within high-income healthcare systems frequently demonstrate substantially reduced performance when applied to geographically distinct populations with different epidemiological characteristics, pathogen distributions, and clinical practices [31]. Furthermore, the limited interpretability of certain deep learning models, often described as “black boxes”, continues to represent an important barrier to widespread clinical implementation, particularly when clinicians are required to understand and explain the rationale underlying algorithm-generated recommendations to patients and colleagues [30].

Consequently, artificial intelligence should not be viewed as a replacement for clinical judgement but rather as an advanced decision-support tool capable of augmenting diagnostic accuracy and improving antimicrobial stewardship when thoughtfully integrated into multidisciplinary clinical practice. However, methodological standards are also lagging behind enthusiasm: most published AMR prediction models are retrospective, single-centre, and internally validated only, and few report according to established prediction-model reporting guidance or undergo prospective external validation in the settings where they would be deployed [28,31]. This gap between technological promise and methodological rigour underscores the need for more robust evaluation frameworks and transparent reporting to ensure that AI tools can be safely and effectively translated into routine clinical care.

A realistic appraisal of where AI can and cannot contribute has been articulated by Arnold and colleagues, who distinguish tasks in which machine learning has already delivered measurable gains, notably susceptibility prediction from structured laboratory data and compound prioritisation in discovery pipelines, from tasks in which performance remains constrained by data rather than by algorithms, such as the prediction of resistance in organisms whose mechanisms are polygenic or expression-dependent [55]. This distinction is a useful corrective to undifferentiated enthusiasm, and we adopt it in the sections that follow.

### 3.2. Genomic Surveillance and Whole-Genome Sequencing

The widespread adoption of whole-genome sequencing (WGS) has profoundly transformed our understanding of antimicrobial resistance at the molecular and epidemiological levels. Traditional culture-based susceptibility testing remains essential for routine clinical management but provides limited information regarding the genetic mechanisms underlying resistance, the evolutionary relationships between bacterial isolates, or the transmission dynamics of resistant clones within healthcare settings and communities [32]. Genomic technologies overcome these limitations by enabling comprehensive characterisation of resistance determinants, mobile genetic elements, virulence factors, and transmission dynamics within a single analytical workflow [33,34].

Beyond identifying known resistance genes through database comparison, WGS allows investigators to reconstruct transmission networks with remarkable precision, facilitating the early detection of hospital outbreaks and the identification of emerging resistant clones before widespread dissemination occurs [36]. This capability has become particularly valuable for high-priority pathogens such as carbapenem-resistant Enterobacterales, multidrug-resistant *Pseudomonas aeruginosa*, and *Acinetobacter baumannii*, where rapid recognition of transmission events is essential for effective infection prevention and control measures [32,34]. The practical value of genomic surveillance depends on the reference databases and analytical pipelines that convert raw sequence data into interpretable resistance and epidemiological information. The Comprehensive Antibiotic Resistance Database (CARD), ResFinder, AMRFinderPlus and the curated reference sets maintained by the National Center for Biotechnology Information provide the resistance-gene ontologies and detection algorithms on which most clinical and public-health genomic workflows rely [56,57,58]. Platform-level infrastructures such as Pathogenwatch and NCBI Pathogen Detection provide shared analytical pipelines, continuously updated clustering of submitted isolates, and cross-border transmission detection without requiring every laboratory to maintain its own bioinformatic stack [59]. The harmonisation of these pipelines across countries, together with agreed minimum reporting sets, is now recognised as a prerequisite for comparable genomic surveillance across jurisdictions.

Increasingly, genomic data are also being incorporated into predictive susceptibility models that aim to infer antimicrobial resistance directly from sequence information. Rather than relying exclusively on time-consuming phenotypic testing, machine learning algorithms can analyse combinations of resistance genes, chromosomal mutations, regulatory pathway alterations, and other genomic features to predict antimicrobial susceptibility with increasing accuracy [28,60]. Performance is, however, strongly organism- and agent-dependent: genotype-based prediction is already reliable where resistance is conferred by a small number of well-characterised determinants, but remains unsatisfactory where resistance is polygenic, expression-dependent, or mediated by efflux and porin changes, as in *Pseudomonas aeruginosa* and for several β-lactam/β-lactamase-inhibitor combinations [34]. These limitations underscore that genotype-based prediction, while promising, is not yet a panacea and must be interpreted alongside phenotypic methods until the underlying biological complexity is better understood.

Despite these remarkable advances, important challenges remain for the widespread implementation of genomic surveillance. Standardisation of sequencing protocols, harmonisation of bioinformatic pipelines, rigorous quality assurance procedures, secure data sharing mechanisms, and adequate computational infrastructure continue to vary substantially across countries and healthcare systems [33]. Addressing these issues through international collaboration and investment will be essential before genomic surveillance can be fully integrated into routine global AMR monitoring programmes [34].

### 3.3. Metagenomic Diagnostics and Culture-Independent Approaches

The inherent limitations of conventional culture-based microbiology, including slow turnaround times, inability to culture fastidious organisms, and reduced sensitivity in patients already receiving antimicrobial therapy, have stimulated growing interest in metagenomic sequencing as a culture-independent diagnostic strategy [32]. Unlike standard microbiological methods that require prior bacterial isolation and pure culture, metagenomics enables simultaneous identification of pathogens and antimicrobial resistance genes directly from clinical specimens without the need for culture amplification. The same culture-independent approaches have been applied to environmental and hospital wastewater phageomes, providing a complementary view of the viral fraction of the resistome [61].

This approach is particularly attractive in several challenging clinical scenarios: patients who have already received antimicrobial therapy (where cultures may be falsely negative), polymicrobial infections (where multiple pathogens may be present), or situations where conventional cultures are slow, insensitive, or unable to identify fastidious organisms [30]. By analysing the complete microbial genetic content of a clinical sample, metagenomic sequencing provides a comprehensive overview of both the infecting organisms and the associated resistome, potentially shortening diagnostic turnaround times from days to hours while substantially expanding the spectrum of detectable pathogens [60].

Although current costs, technical complexity, and challenges in data interpretation continue to limit widespread implementation in routine clinical practice, ongoing technological improvements are progressively reducing these barriers. Advances in portable sequencing platforms, automated bioinformatic pipelines, and cloud-based computational resources are making metagenomic approaches increasingly accessible to clinical laboratories [32]. Nevertheless, interpretation remains the principal obstacle rather than sequencing itself: detection of a resistance gene in a metagenome does not establish which organism carries it, whether it is expressed, or whether it is clinically relevant, and the field still lacks agreed reporting thresholds, quality metrics, and external quality assessment schemes [32,60]. These interpretative challenges underscore the need for continued development of bioinformatic tools, curated reference databases, and standardised reporting guidelines before metagenomics can be reliably integrated into routine diagnostic workflows.

### 3.4. Digital Epidemiology and Predictive Surveillance

Traditional antimicrobial resistance surveillance has historically relied on retrospective reporting of laboratory-confirmed cases, often with substantial delays between data collection and public health action. While essential for monitoring long-term epidemiological trends, this approach frequently delays recognition of emerging resistance patterns and limits opportunities for early intervention [7]. Digital surveillance seeks to overcome these limitations by integrating multiple data streams, including microbiological databases, electronic health records, antimicrobial consumption data, environmental monitoring, population mobility patterns, and genomic surveillance, into continuously updated analytical platforms capable of providing real-time situational awareness [26,36].

Artificial intelligence substantially enhances the value of these surveillance systems by identifying subtle epidemiological signals that may precede measurable increases in resistance prevalence [62]. Predictive models can estimate the probability of future resistance emergence within specific healthcare facilities, geographical regions, or patient populations, allowing earlier implementation of targeted infection prevention measures and antimicrobial stewardship interventions [28,30].

The WHO GLASS programme has demonstrated the feasibility of coordinated international AMR surveillance, establishing standardized protocols and reporting mechanisms that facilitate global comparisons [7]. Recent innovations increasingly incorporate genomic data and advanced computational modelling into surveillance frameworks, enabling more precise tracking of resistance emergence and spread. Rather than simply documenting resistance after it has emerged, future surveillance systems aim to provide real-time situational awareness capable of informing both clinical decision-making and public health policy at local, national, and global levels [26].

Importantly, surveillance is also expanding beyond traditional hospital and community settings. Environmental monitoring of wastewater, surface water, agricultural settings, and animal reservoirs is becoming an increasingly valuable source of information regarding the circulation of resistance genes across the broader One Health ecosystem [32]. Wastewater surveillance, in particular, has demonstrated the potential to detect changes in resistance patterns before corresponding increases become evident within healthcare facilities, providing an early warning system that could inform public health responses [62].

At the European level, integrated reporting across sectors has been institutionalised through the joint analyses periodically produced by ECDC, EFSA and EMA, which bring together data from EARS-Net (clinical AMR), ESVAC (veterinary antimicrobial consumption) and the food-chain surveillance networks into a single integrated analysis (JIACRA). The most recent JIACRA report provides one of the few examples in which human, animal and food-chain data on antimicrobial consumption and resistance are analysed jointly rather than described as separately integrable [63].

At the global level, the Global Sewage Surveillance Project has demonstrated the feasibility of standardised metagenomic sampling across dozens of countries and has provided the methodological template for national wastewater networks that were subsequently repurposed after the SARS-CoV-2 pandemic [64].

Finally, surveillance prioritisation has been increasingly informed by the WHO Bacterial Priority Pathogens List, whose 2024 update explicitly identifies the pathogen–antibiotic combinations for which new surveillance, diagnostics and therapeutics are most urgently needed, and provides a coordinating instrument for national and international surveillance investments [65].

### 3.5. Rapid Diagnostics and Point-of-Care Testing

Rapid diagnosis remains one of the greatest unmet needs in antimicrobial stewardship and infection management. Every hour of delay in identifying the causative pathogen and its susceptibility profile may increase inappropriate antimicrobial exposure, while unnecessary empirical broad-spectrum therapy contributes directly to resistance selection and adverse patient outcomes [30].

Recent advances in artificial intelligence are accelerating diagnostic workflows through automated interpretation of microbiological data, optimisation of laboratory processes, and integration with molecular diagnostic platforms [31]. Machine learning algorithms can analyse complex laboratory datasets, including matrix-assisted laser desorption/ionization time-of-flight (MALDI-TOF) mass spectrometry spectra, growth curve characteristics, and biochemical reaction patterns, to predict antimicrobial susceptibility before conventional phenotypic testing has been completed, thereby substantially reducing the time required to initiate targeted therapy [28].

Beyond MALDI-TOF-based machine learning, several point-of-care and near-patient platforms are now in various stages of clinical evaluation. Syndromic multiplex PCR panels (e.g., BioFire FilmArray Blood Culture Identification 2) identify pathogens and key resistance markers directly from positive blood cultures within approximately one hour [66]. Direct-from-blood-culture rapid AST systems (e.g., Accelerate Pheno, Alfred 60AST) provide phenotypic susceptibility results hours earlier than conventional workflows [67]. Isothermal amplification approaches, including loop-mediated isothermal amplification (LAMP) and recombinase polymerase amplification (RPA), enable low-complexity, instrument-light detection of resistance genes at the point of care [68], and CRISPR-based diagnostics have advanced from proof-of-concept to early clinical evaluation [69]. Host-response markers, particularly procalcitonin and emerging transcriptomic signatures of bacterial versus viral infection, have been evaluated as tools to reduce unnecessary antibiotic initiation in respiratory infections and sepsis [70].

A consistent finding across this literature is that the clinical benefit of rapid diagnostics is conditional rather than automatic. Randomised and quasi-experimental studies have repeatedly shown that time-to-optimal-therapy improves only when rapid diagnostics are coupled to a structured antimicrobial stewardship intervention, including real-time alerting, audit-and-feedback, and prescriber education [71,72]. Where such coupling is absent, rapid tests frequently fail to alter prescribing behaviour and may even increase broad-spectrum use when results are misinterpreted or acted upon without stewardship support. This finding is directly relevant to the surveillance argument developed in this review: diagnostic technologies and stewardship programmes are complementary components of a single intervention, not independent innovations, and their standalone value should not be overstated.

Parallel developments in CRISPR-based molecular diagnostics offer an additional opportunity to improve rapid pathogen detection and resistance gene identification. These highly sensitive and specific assays can identify specific resistance genes within a matter of hours, directly from clinical specimens, and may eventually support point-of-care testing for clinically relevant resistant pathogens [69]. Although widespread clinical implementation remains limited by regulatory, cost, and workflow considerations, early studies suggest that CRISPR diagnostics could significantly shorten the interval between specimen collection and targeted antimicrobial treatment, potentially improving patient outcomes while reducing unnecessary antibiotic exposure.

The combination of rapid molecular diagnostics with AI-assisted interpretation represents one of the most promising developments in precision infectious diseases, allowing clinicians to move from empirical treatment strategies toward truly individualised antimicrobial therapy based on real-time pathogen identification and susceptibility prediction. This promise, however, is realised only when the diagnostic result is coupled to a stewardship response capable of acting on it.

### 3.6. AI in Antimicrobial Drug Discovery

Perhaps the most transformative application of artificial intelligence in the fight against antimicrobial resistance lies in antimicrobial drug discovery. The traditional development of novel antibiotics is notoriously costly, time-consuming, and associated with extremely high failure rates, with average timelines exceeding a decade and costs approaching or exceeding one billion dollars per successful candidate [73]. Artificial intelligence is beginning to reshape this process by rapidly screening millions of chemical structures, predicting antimicrobial activity, identifying potential toxicity profiles, and optimising candidate molecules before any laboratory synthesis occurs [29,37].

Recent breakthroughs in generative deep learning have demonstrated that AI can design entirely novel antimicrobial compounds rather than merely identifying previously existing molecules from chemical libraries [30]. In a landmark study, Krishnan and colleagues used generative deep learning to create de novo antibiotics with potent activity against multidrug-resistant bacterial pathogens, illustrating how computational models may substantially accelerate the antibiotic development pipeline and potentially overcome the limitations of traditional discovery approaches [74].

Beyond molecule generation, AI could also facilitate drug repurposing (identifying existing drugs with previously unrecognised antimicrobial activity), prediction of synergistic antimicrobial combinations, optimisation of pharmacodynamic properties, and prioritisation of compounds for experimental validation [30]. These capabilities may significantly reduce both development costs and timelines, helping to revitalise a field that has experienced declining pharmaceutical investment over recent decades due to unfavourable economic incentives and scientific challenges [37,73]. It should be emphasised, however, that the principal bottleneck in the antibacterial pipeline is economic rather than computational. Because new antibiotics are deliberately held in reserve, they cannot recover their development costs through sales volume, and several companies that successfully brought novel agents to market subsequently became insolvent. Pull incentives that delink revenue from consumption, including the subscription-style payment models piloted in the United Kingdom and Sweden and the transferable exclusivity vouchers adopted by the European Union, are therefore at least as important to the pipeline as any algorithmic advance [75,76,77,78]. To date, the contribution of AI to antibacterial discovery also remains largely preclinical, and candidate molecules generated by these methods have yet to demonstrate efficacy and safety in humans [79].

Laxminarayan and colleagues set out the corresponding agenda in detail, arguing that antibiotic development, vaccine deployment and diagnostic access must be expanded together, and that measures addressing any one of the three in isolation will not alter the trajectory of resistance [80]. Their analysis also makes clear that access and stewardship are not competing objectives: in settings where a substantial proportion of deaths from resistant infection are attributable to the unavailability of effective agents, the binding constraint on the pipeline is distributional as much as scientific.

Although experimental validation remains absolutely indispensable for confirming computational predictions, artificial intelligence is rapidly becoming an essential component of next-generation antimicrobial discovery. Rather than replacing laboratory research, AI enables investigators to focus experimental resources on the most promising candidates, substantially increasing the efficiency and success rate of the drug development process while reducing the time and cost required to bring new antimicrobial agents to clinical use.

### 3.7. Operational Implementation: What Is Actually Deployed

The preceding subsections describe what integrated, AI-enabled AMR surveillance could achieve. It is equally important to state what is currently in operation, because the gap between the two is considerable and is where most of the practical difficulty lies.

At the level of routine international surveillance, the WHO GLASS programme and, within Europe, the ECDC EARS-Net network collect standardised phenotypic data from national reference systems; the periodic joint inter-agency analyses of antimicrobial consumption and resistance produced by ECDC, EFSA and EMA represent one of the few examples in which human, animal and food-chain data are actually analysed in the same document rather than merely described as integrable. The Danish DANMAP programme remains the most frequently cited national model of genuinely integrated human–animal–food reporting, and it is instructive that it rests on decades of institutional investment, a small and well-connected set of laboratories, and national identifiers that permit linkage, conditions that are not readily transferable. At the genomic level, platforms such as Pathogenwatch and NCBI Pathogen Detection provide shared analytical infrastructure and continuously updated clustering of submitted isolates, and have been used to identify cross-border transmission of high-risk clones. In the environmental compartment, the Global Sewage Surveillance Project demonstrated the feasibility of comparable metagenomic sampling across dozens of countries, and several national wastewater networks established for SARS-CoV-2 monitoring have subsequently been extended to resistance genes. At the clinical level, the most mature AI applications are narrow rather than integrative: decision-support tools that predict the probability of resistance for an individual patient from local epidemiology, prior exposure and routine laboratory variables, and rule-based or model-assisted stewardship systems embedded in electronic prescribing.

**Figure 2:**
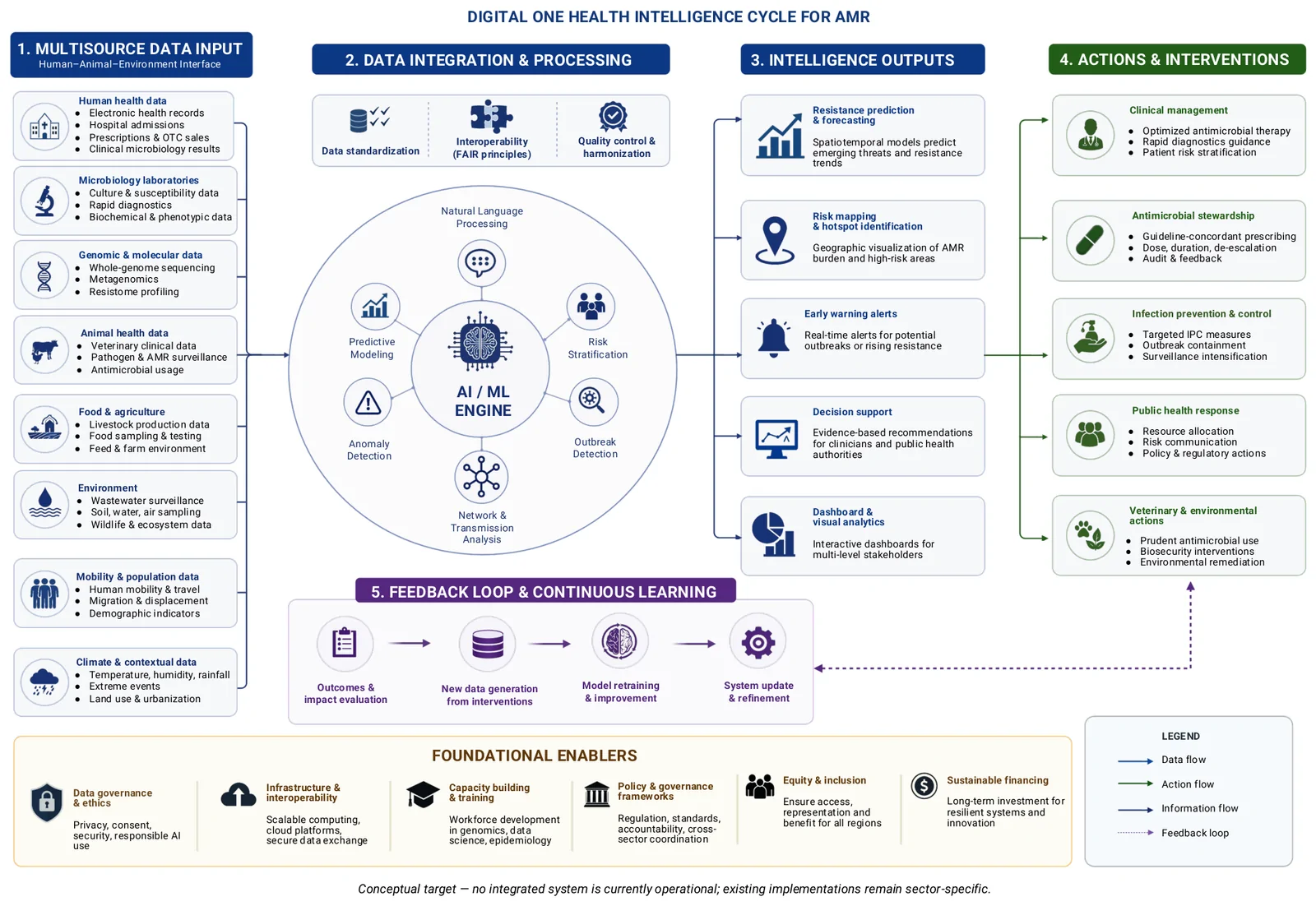
**Conceptual framework for a digital One Health intelligence cycle for AMR.** The framework illustrates how multisource data generated across human health, animal health, food systems, environmental surveillance, mobility, climate, and genomics could be integrated through interoperable digital infrastructures and analysed using AI and ML. This process generates predictive intelligence, including resistance forecasting, hotspot identification, outbreak detection, early-warning alerts, and clinical decision support. These outputs inform coordinated interventions across clinical care, antimicrobial stewardship, infection prevention and control, public health, veterinary medicine, and environmental health. Continuous feedback from surveillance outcomes and implemented interventions enables iterative model refinement, creating a dynamic learning system that supports proactive, rather than reactive, antimicrobial resistance preparedness within a One Health framework. No component of this integrated cycle is currently operational as a single system; existing implementations are sector-specific and are described in Section 3.7. The figure is therefore presented as a conceptual target to guide incremental interoperability and future integration.

What these examples have in common is that each operates within a single sector or a single data type. To our knowledge, no system currently integrates clinical, veterinary, food-chain, environmental, genomic and climatic data into a single continuously updated predictive platform of the kind described in Figure 2, which we therefore present as a conceptual target rather than as a description of existing practice. The obstacles encountered by the implementations listed above are consistent and worth stating plainly.

First, the sectors do not share data models. Human clinical data are increasingly standardised, veterinary and food-chain data are collected under different regulatory obligations with different granularity, and environmental metagenomic output has no equivalent of a patient record at all; harmonisation is therefore a semantic problem, not merely a technical one. Second, the legal basis for cross-sector linkage is frequently absent, and data protection, commercial confidentiality in food production, and national reluctance to share resistance data with trade implications all restrict the flow of information that integrated models require. Third, the outcome labels used to train predictive models are unstable, because clinical breakpoints are periodically revised, so that identical minimum inhibitory concentrations may be classified differently across the time span of a training dataset. Fourth, environmental surveillance lacks a defensible denominator: the detection of a resistance gene in a wastewater catchment is not readily convertible into a population prevalence, and sampling points are typically chosen for accessibility rather than representativeness. Fifth, almost all published models are retrospective, single-centre and internally validated; prospective external validation in the settings where deployment is proposed remains rare, and reporting according to established prediction-model guidance is inconsistent. Sixth, the sustainability of these systems is largely project-dependent, and several promising integrated pilots have not survived the end of their funding cycles.

A further set of practical barriers concerns data governance and analytical architecture. Cross-sector integration of AMR data requires clear rules on data ownership, consent, and secondary use, particularly when human clinical data are linked to veterinary, environmental, or commercial food-chain data. Privacy-preserving architectures, including federated learning approaches in which models are trained across institutions without moving the underlying data, have been proposed as a technical solution to this problem. Federated learning allows participating laboratories and hospitals to contribute to a shared predictive model without disclosing patient-level records, and pilot implementations in clinical microbiology and genomic surveillance have demonstrated feasibility, though at the cost of increased coordination and infrastructure requirements [81]. Standardisation of data models and terminologies, including minimum reporting sets, agreed metadata standards, and shared identifiers, is a prerequisite for any of these approaches to scale beyond pilot studies. Without such standardisation, the interoperability required for integrated One Health intelligence remains aspirational rather than operational.

The practical implication is that progress is more likely to come from incremental interoperability, for example shared identifiers, common metadata standards, agreed minimum reporting sets, and federated analysis that leaves data in place, than from the construction of a single central platform. This more modest framing is consistent with the conclusions of recent syntheses of AI applications in One Health AMR work, which report a literature dominated by proof-of-concept studies and a marked scarcity of deployed, evaluated systems [45,46], and with reviews of AI-integrated biosensing, where analytical performance is often excellent under laboratory conditions but field validation and integration into reporting workflows remain the limiting steps [47].

Taken together, artificial intelligence, genomics, metagenomics, digital surveillance, predictive analytics, and computational drug discovery should not be viewed as independent innovations but rather as interconnected components of a single, synergistic technological ecosystem. Their true potential lies in thoughtful integration and interoperability: genomic surveillance informs predictive models; AI enhances diagnostic interpretation and clinical decision-making; digital surveillance guides public health interventions; and computational platforms accelerate therapeutic development [30,32]. Collectively, these advances are transforming antimicrobial resistance management from a reactive discipline centred on treatment failure and outbreak response into a proactive strategy focused on early detection, accurate prediction, and effective prevention.

This technological revolution, however, addresses primarily the detection and monitoring dimensions of the AMR challenge. Even with increasingly sophisticated diagnostic and surveillance tools, sustainable control of antimicrobial resistance will also require innovative therapeutic approaches capable of reducing dependence on conventional antibiotics and expanding the treatment options available for multidrug-resistant infections. The integration of these technologies into a continuous Digital One Health Intelligence Cycle is illustrated in Figure 2, which proposes a framework for linking data collection, analysis, and intervention across human, animal, and environmental sectors to support proactive AMR preparedness.

A consolidated overview of the surveillance, diagnostic and therapeutic technologies discussed in Section 3 and Section 4, including their current stage of development or implementation, principal advantages, principal limitations and level of translational maturity, is provided in Table 1.

## 4. Therapeutic Innovations for AMR: Beyond Conventional Antibiotics

Although technological innovation is revolutionising the detection and prediction of antimicrobial resistance, surveillance alone cannot solve the growing shortage of effective therapeutic options. The continuous emergence of multidrug-resistant pathogens, coupled with the limited development of novel antibiotic classes over the past decades, has highlighted the urgent need for alternative antimicrobial strategies capable of reducing dependence on conventional antibiotics [2]. The antibacterial pipeline has remained alarmingly sparse, with few new classes reaching the clinic in recent years, while resistance continues to expand across virtually all pathogen families [3,73].

Unlike previous decades, when the discovery of new antibacterial molecules was considered the primary solution to resistance, current research increasingly focuses on complementary approaches that either directly target bacterial pathogens through non-conventional mechanisms or enhance host defence while minimising selective pressure for resistance [2]. Rather than replacing antibiotics altogether, these emerging strategies are expected to become part of combination therapeutic approaches tailored to specific pathogens, resistance mechanisms, and clinical scenarios, offering a more diversified and resilient arsenal against resistant infections.

### 4.1. Bacteriophage Therapy for Multidrug-Resistant Infections

Bacteriophages, that is, viruses that selectively infect and replicate within bacterial cells, represent one of the oldest antimicrobial strategies, first described in the early twentieth century, and are currently experiencing a renewed resurgence of interest [79]. Their ability to specifically lyse bacterial cells while preserving the commensal microbiota offers an important theoretical advantage over broad-spectrum antibiotics, which indiscriminately disrupt the entire microbial ecosystem [2]. Furthermore, phages can co-evolve alongside their bacterial hosts through natural selection, potentially limiting the long-term development of resistance and offering a dynamic, adaptive therapeutic approach [79].

Recent advances in genomic engineering, synthetic biology, and personalised phage selection have substantially improved the feasibility and scalability of clinical phage therapy [79]. High-throughput screening methods now enable rapid identification of phages with optimal lytic activity against specific bacterial strains, while advances in phage formulation and delivery have addressed some of the practical challenges that previously limited clinical application. Compassionate-use experiences and early-phase clinical studies have reported encouraging outcomes in patients with otherwise untreatable multidrug-resistant infections, including those caused by carbapenem-resistant *Pseudomonas aeruginosa*, *Acinetobacter baumannii*, and *Staphylococcus aureus* [79,82,83,84]. Notably, phage therapy has shown particular promise in chronic biofilm-associated infections, such as those complicating prosthetic joint infections, cystic fibrosis pulmonary exacerbations, and chronic wound infections, where conventional antibiotics frequently fail due to poor biofilm penetration and the presence of metabolically dormant persister cells.

Nevertheless, important challenges remain for the widespread clinical adoption of phage therapy. Narrow host specificity, each phage typically targets a limited range of bacterial strains, necessitates careful matching of phages to individual patient isolates, which can be logistically demanding and time-consuming [79]. Manufacturing standardisation and quality control present additional hurdles, as phage preparations are biological products with inherent variability that complicate regulatory approval. Moreover, the possibility of bacterial resistance to therapeutic phages, while potentially addressable through phage cocktail formulations or sequential therapy, remains a concern that requires careful monitoring and adaptive strategies [79].

Despite these limitations, phage therapy is increasingly viewed not as a replacement for antibiotics but as a complementary strategy with a distinct role in the therapeutic armamentarium. Its particular value lies in chronic, biofilm-associated infections and extensively drug-resistant pathogens where conventional options have been exhausted, and its integration with antibiotics may offer synergistic benefits through combined bactericidal and anti-biofilm activity. Nevertheless, randomised controlled evidence remains scarce, and the few controlled trials completed to date have produced mixed results [82,83,84], underlining that compassionate-use case series cannot substitute for controlled evaluation and that regulatory pathways for personalised biological products are still being defined. These evidentiary and regulatory gaps highlight the urgent need for well-designed clinical trials, standardised manufacturing protocols, and adaptive regulatory frameworks that can accommodate the unique characteristics of phage-based therapies.

### 4.2. CRISPR-Based Antimicrobials for Precision Gene Targeting

The development of CRISPR-Cas technologies has introduced the possibility of highly targeted antimicrobial interventions that operate at the genetic level with unprecedented precision [85]. Unlike conventional antibiotics, which indiscriminately affect susceptible bacterial populations and exert broad selective pressure on the microbiome, CRISPR-based systems can selectively eliminate bacteria carrying specific resistance genes or virulence determinants while preserving non-target microorganisms. This precision approach represents a fundamental departure from traditional antimicrobial strategies, offering the potential for pathogen-specific eradication without collateral damage to the commensal microbiota [85,86,87,88].

Experimental studies have demonstrated that CRISPR constructs delivered through bacteriophages or conjugative plasmids can specifically remove resistance-conferring genetic elements, including carbapenemase-encoding plasmids, extended-spectrum β-lactamase genes, and other mobile genetic elements that drive the horizontal dissemination of resistance [85,86,87]. The most informative recent demonstration is that of Gencay and colleagues, who engineered phage vectors carrying antibacterial CRISPR–Cas payloads and showed selective reduction of targeted *Escherichia coli* populations in the murine gut while leaving non-target members of the microbiota substantially intact [88]. That study is valuable precisely because it illustrates both the promise and the present limits of the approach: efficacy depended on delivery efficiency and on host range rather than on the nuclease itself, and the reductions achieved in vivo were partial rather than absolute. It therefore substantiates, with a concrete example, the characterisation of this technology as promising but still preclinical.

Although these technologies remain largely at the preclinical stage, they represent one of the most promising examples of precision antimicrobial therapy currently under development. Their potential is particularly compelling when integrated with rapid molecular diagnostics capable of identifying specific resistance determinants before treatment initiation, enabling truly personalised antimicrobial interventions guided by the genomic profile of the infecting organism [85].

It is important to distinguish therapeutic CRISPR approaches from the CRISPR-based diagnostic platforms discussed in Section 3.5. While diagnostic applications aim to rapidly identify resistant pathogens through nucleic acid detection, therapeutic CRISPR seeks to directly eliminate or genetically disable them, representing a fundamentally different application of the same underlying technology [85].

### 4.3. Antimicrobial Peptides in Host Defence and Infection Control

Antimicrobial peptides (AMPs) constitute an evolutionarily conserved component of innate immunity found across all multicellular organisms, and they have attracted growing attention as potential alternatives to traditional antibiotics [89]. Their mechanisms of action are notably diverse, often involving disruption of bacterial membranes through electrostatic interactions, intracellular targeting of essential processes, immunomodulation of host responses, and interference with biofilm formation. This multi-targeted mode of action makes the emergence of resistance comparatively less frequent than with conventional antimicrobial agents, which typically act through single, specific molecular targets [89,90].

Several naturally occurring and synthetic AMPs have demonstrated broad-spectrum activity against ESKAPE pathogens, a group of highly virulent and drug-resistant bacteria that are leading causes of healthcare-associated infections worldwide, including strains resistant to multiple antibiotic classes [89]. In addition to their direct antimicrobial effects, many peptides exert potent anti-inflammatory and wound-healing properties through modulation of host immune responses, suggesting potential applications in complex infections where excessive host inflammation contributes to tissue damage and impaired healing [89].

Despite promising preclinical results and the successful clinical application of some AMPs in topical formulations, systemic clinical translation has been substantially slowed by several significant challenges [89]. These include dose-limiting toxicity to host cells, particularly nephrotoxicity and haemolytic activity; rapid proteolytic degradation in biological fluids, leading to short half-lives and unfavourable pharmacokinetic profiles; and high production costs that limit scalability and commercial viability. Ongoing advances in peptide engineering and computational design, including AI-assisted optimisation of peptide sequences to enhance stability, reduce toxicity, and improve pharmacokinetic properties, may help overcome many of these limitations, illustrating the growing convergence between biotechnology and artificial intelligence [89,90,91].

Recent work also indicates that the therapeutic value of AMPs may lie as much in combination as in monotherapy. Zhang and colleagues described a multifunctional peptide that combines cell-penetrating properties with direct antibacterial activity and with potentiation of β-lactams, restoring activity against methicillin-resistant *Staphylococcus aureus* and carbapenem-resistant Enterobacteriaceae in preclinical models [91]. Peptides acting as adjuvants rather than as stand-alone agents may permit lower doses, which is directly relevant to the toxicity constraints described below.

### 4.4. Nanomedicine for Targeted Antimicrobial Delivery

Nanotechnology offers innovative opportunities to enhance antimicrobial therapy by improving drug delivery, increasing local antibiotic concentrations at sites of infection, and reducing systemic toxicity through targeted release mechanisms [92]. Nanoparticles can function as antimicrobial agents themselves, through mechanisms such as membrane disruption, reactive oxygen species generation, and metal ion release, or as sophisticated carriers capable of delivering antibiotics directly to sites of infection, thereby improving pharmacokinetic performance while reducing off-target effects and associated toxicity [92,93].

Several classes of nanomaterials, including liposomes, polymeric nanoparticles, metallic nanoparticles (particularly silver, gold, and zinc oxide), and hybrid nanostructures, have demonstrated activity against resistant bacteria through diverse mechanisms [92]. These approaches may prove particularly valuable for biofilm-associated infections, where conventional antibiotics frequently exhibit limited penetration and reduced efficacy due to the protective extracellular matrix and metabolically dormant bacterial subpopulations present within biofilms [92].

Although most nanomedicine applications remain under clinical investigation, they illustrate how engineering approaches may effectively complement pharmacology in addressing antimicrobial resistance. The ability to encapsulate multiple antimicrobial agents within a single nanoparticle formulation, achieving synergistic effects and reducing the likelihood of resistance emergence, represents a particularly promising direction. Additionally, stimuli-responsive nanoparticles that release their payload in response to specific bacterial enzymes or environmental pH changes offer the potential for highly localised therapy with minimal systemic exposure [92,93].

### 4.5. Microbiota-Based Therapies for Microbiome Restoration

Increasing recognition of the human microbiome as a critical regulator of colonisation resistance against pathogenic bacteria has stimulated growing interest in microbiota-directed interventions as a strategy for combating antimicrobial resistance [94]. Broad-spectrum antibiotic exposure frequently disrupts the intestinal microbial ecosystem, reducing microbial diversity and creating ecological niches that facilitate colonisation by multidrug-resistant organisms, including vancomycin-resistant enterococci, carbapenem-resistant Enterobacterales, and *Clostridioides difficile* [94].

Strategies aimed at restoring microbial homeostasis, including faecal microbiota transplantation (FMT), live biotherapeutic products containing defined bacterial consortia, and next-generation probiotics engineered with specific functional properties, have demonstrated encouraging results in reducing intestinal carriage of resistant pathogens in selected patient populations [94]. FMT, in particular, has shown substantial efficacy in restoring microbial diversity and eliminating colonisation by multidrug-resistant organisms, although the evidence remains heterogeneous and largely derived from small case series and uncontrolled studies [94,95,96].

While the evidence base remains heterogeneous and standardisation challenges persist, microbiota restoration represents a promising adjunctive strategy for limiting the ecological consequences of prolonged antibiotic exposure and reducing the reservoir of resistant organisms that can seed subsequent infections [94]. Rather than directly targeting pathogens, microbiome-based therapies seek to strengthen the natural ecological barriers that normally prevent colonisation by resistant microorganisms, offering a fundamentally different approach to resistance control that addresses the ecosystem rather than the individual pathogen [95,96].

It is worth noting that microbiota-directed approaches are not confined to human medicine: competitive exclusion products administered to poultry to reduce *Salmonella* and *Campylobacter* colonisation are an established veterinary application of the same ecological principle, and predate most of the human evidence base.

### 4.6. Host-Directed Immunotherapy for Adjunctive Infection Management

An emerging concept in infectious diseases is that successful treatment may not always require direct bacterial killing [97]. Host-directed therapies aim to enhance protective immune responses or modulate harmful inflammation, thereby improving infection control while simultaneously reducing the selective pressure that drives antimicrobial resistance [98]. By targeting host pathways rather than bacterial targets, these approaches theoretically avoid the emergence of bacterial resistance and may offer durable benefits across diverse pathogen populations [98].

The rationale for host-directed immunotherapy is grounded in the recognition that the host immune response plays a critical role in determining the outcome of bacterial infections. In many cases, morbidity and mortality are driven not only by bacterial burden but also by dysregulated inflammation, tissue damage, and immune exhaustion. By modulating these host factors, immunotherapy may improve outcomes even when antibiotics are unable to achieve complete bacterial clearance [98].

Potential approaches include monoclonal antibodies targeting bacterial toxins or surface antigens, which can neutralise virulence factors and facilitate opsonophagocytic killing; cytokine modulation to enhance or dampen immune responses appropriately, including the use of interferons, interleukins, and checkpoint inhibitors; enhancement of innate immune responses such as phagocytosis, neutrophil activity, and natural killer cell function; and immunometabolic interventions that optimise the host’s metabolic state to support effective immune function [97,98].

Although currently limited to selected pathogens and clinical settings, notably, monoclonal antibodies against *Clostridioides difficile* toxins (bezlotoxumab), immunomodulatory agents in tuberculosis, and adjunctive corticosteroids in severe infections, these strategies may become increasingly important as adjunctive therapies for severe multidrug-resistant infections where antimicrobial options are limited [97,98]. The development of novel immunomodulatory agents, including small molecules targeting specific immune checkpoints and biologics designed to enhance antimicrobial host defence, represents an active area of research with significant potential for clinical translation.

Importantly, host-directed therapies are unlikely to replace antibiotics but may substantially improve therapeutic outcomes when combined with pathogen-directed antimicrobial treatment. By reducing the bacterial burden through enhanced host clearance, these approaches may lower the required dose and duration of antibiotics, potentially reducing selective pressure for resistance while improving clinical outcomes in severe infections [98]. Moreover, the combination of host-directed and pathogen-directed therapies may offer synergistic benefits, particularly in infections caused by intracellular pathogens or in immunocompromised patients where host defence is already compromised.

### 4.7. Photodynamic Therapy for Localised Antimicrobial Treatment

Photodynamic therapy (PDT) represents another innovative non-antibiotic strategy based on the activation of photosensitising molecules by specific wavelengths of light, leading to the local generation of reactive oxygen species capable of destroying microorganisms through oxidative damage to multiple cellular targets [99]. Because oxidative damage simultaneously affects multiple bacterial targets, including membranes, proteins, and nucleic acids, the development of resistance appears considerably less likely than with conventional antibiotics that act through specific molecular targets [99,100].

The mechanism of PDT involves three essential components: a photosensitiser, light of an appropriate wavelength, and molecular oxygen. Upon light activation, the photosensitiser transfers energy to molecular oxygen, generating highly reactive singlet oxygen and other reactive oxygen species that cause oxidative damage to bacterial cells [99]. This multi-targeted mechanism is particularly advantageous against biofilms, where conventional antibiotics often fail due to limited penetration and the presence of metabolically dormant persister cells. Moreover, PDT can also exert immunomodulatory effects, promoting wound healing and reducing inflammation, which further contributes to its therapeutic potential [99].

PDT has shown particular promise for superficial infections, chronic wounds, dental infections, and biofilm-associated diseases, where local light delivery is technically feasible and the tissue penetration of light is sufficient for effective activation [99]. The approach has been successfully applied in dermatological infections (including acne, fungal infections, and cutaneous leishmaniasis), periodontal disease, and infected chronic ulcers, with encouraging results in reducing bacterial burden and promoting wound healing [99,100]. In the context of antimicrobial resistance, PDT offers the additional advantage of being effective against multidrug-resistant pathogens, including MRSA, carbapenem-resistant *P. aeruginosa*, and *A. baumannii*, with minimal risk of resistance development.

Although systemic applications remain limited due to challenges in photosensitiser delivery and light penetration through tissues, continued improvements are addressing these barriers [99]. Advances in photosensitiser design, including the development of targeted delivery systems, enhanced photochemical properties, and photosensitisers activated by near-infrared light with better tissue penetration, are expanding the potential applications of PDT. Similarly, innovations in light-delivery systems, such as fibre-optic devices, implantable light sources, and wearable light-emitting patches, are enabling PDT in deeper tissues and more complex anatomical sites [99]. The combination of PDT with conventional antibiotics has shown synergistic effects in some studies, suggesting potential for integrated therapeutic approaches that combine the rapid bactericidal activity of PDT with the sustained antimicrobial effects of antibiotics.

### 4.8. Vaccines and Prevention as Underused Antimicrobial Resistance Interventions

Discussions of the alternatives to antibiotics tends to concentrate on therapeutics, yet the intervention with the strongest existing evidence base for reducing antimicrobial consumption and resistance is preventive rather than curative. Vaccination reduces the incidence of infections that would otherwise be treated with antibiotics, reduces febrile episodes that trigger empirical prescribing, and, in the case of conjugate vaccines, exerts direct selective pressure against resistant serotypes. Pneumococcal conjugate vaccines have been followed by measurable declines in penicillin-non-susceptible invasive disease, typhoid conjugate vaccines address a pathogen in which fluoroquinolone and azithromycin resistance are expanding rapidly, and influenza vaccination reduces inappropriate antibacterial prescribing for viral respiratory illness [101]. Modelling studies suggest that a substantial fraction of the annual burden of bacterial AMR deaths could be averted through wider deployment of existing and pipeline vaccines [101]. Comparable arguments apply to water, sanitation and hygiene (WASH) infrastructure and to infection prevention and control (IPC) programmes, both of which are explicit targets of the 2024 United Nations Political Declaration [54] and of the updated WHO Global Action Plan 2026–2036 [102].

This preventive dimension is not peripheral to the technological agenda described above; it is the setting in which that agenda has to operate. The updated WHO Global Action Plan on Antimicrobial Resistance 2026–2036 is explicitly prevention-first, and the surveillance and diagnostic capabilities discussed in Section 3 and Section 5 are precisely what is required to measure whether preventive interventions are working. A review of emerging strategies that omitted vaccination would therefore give a distorted picture of where the greatest near-term gains are likely to be found [54,101,102].

### 4.9. Distribution of Non-Antibiotic Strategies Across the One Health Compartments

The strategies described above are frequently presented as if they were exclusively human therapeutics, which misrepresents both their current use and their regulatory maturity. Several are already deployed outside human medicine, and in some cases the non-human application is considerably more advanced than the clinical one.

Bacteriophages are the clearest example. Phage preparations targeting *Listeria monocytogenes* have been authorised for application to ready-to-eat foods and food-contact surfaces, and phage products are used in aquaculture and in livestock production to reduce colonisation by specific pathogens [79,82]. In human medicine, by contrast, phage therapy remains largely confined to compassionate use and early-phase trials. The same biological agent therefore occupies opposite ends of the regulatory spectrum depending on the compartment in which it is applied, which offers an unusual opportunity: food-chain and veterinary experience provides manufacturing, stability and host-range data of direct relevance to clinical translation.

Antimicrobial peptides are under development both as human therapeutics and as feed additives intended to replace antibiotic growth promoters in livestock [89,90], the latter being an application at far larger volumes than any clinical use. Nanomaterials with antimicrobial activity are used in water treatment, food packaging and surface disinfection, as well as in experimental drug delivery. Microbiota-directed interventions, as noted above, have an established veterinary form in competitive exclusion products. Photodynamic approaches are applied in dentistry and dermatology in humans, and have been investigated for surface decontamination in food processing and for the treatment of aquaculture pathogens. CRISPR-based antimicrobials remain preclinical in all compartments, although the agricultural interest in targeted decolonisation of food-producing animals is explicit. Vaccination, discussed in Section 4.8, is the intervention with the longest-standing and highest-volume application across human, companion-animal and food-producing-animal compartments simultaneously.

This distribution carries a warning that follows directly from the One Health framework developed in Section 5. The history of colistin demonstrates that the large-scale deployment of an antimicrobial in animal production can select for transferable resistance that subsequently compromises the same agent in human medicine [38,40]. The same logic applies to phages, peptides and microbiota-directed products: selecting for phage resistance in food-production environments, or for cross-resistance between agricultural peptides and host defence peptides, would undermine the clinical utility of the corresponding human therapeutics. Cross-compartment deployment of these strategies should therefore be accompanied from the outset by the integrated resistance monitoring described in Section 5.2, rather than being retrofitted once resistance has emerged.

The future of antimicrobial therapy is unlikely to depend on a single breakthrough technology or a single class of therapeutic agents. Instead, it will probably involve the rational integration of conventional antibiotics with innovative therapeutic platforms, tailored to individual pathogens, resistance mechanisms, and host characteristics [2,73]. Artificial intelligence may assist in selecting optimal treatment combinations based on genomic and phenotypic data; genomics may identify actionable resistance determinants to guide targeted interventions; while phages, CRISPR-based approaches, antimicrobial peptides, microbiota modulation, nanomedicine, and host-directed therapies collectively expand the therapeutic arsenal beyond traditional antibiotics [2]. Rather than signalling the end of the antibiotic era, these innovations point toward a more diversified and resilient therapeutic landscape in which antibiotics become one component of a broader, integrated strategy against antimicrobial resistance [3].

The successful implementation of these diverse approaches, however, will ultimately depend on their incorporation into a coordinated framework that addresses the interconnected nature of human, animal, and environmental health. The principal emerging non-antibiotic therapeutic strategies discussed in this section, including their mechanisms of action, major advantages, current limitations, and stage of clinical development, are summarized in Table 2. This overview highlights the diversity of approaches currently under investigation and their complementary roles in addressing the growing challenge of antimicrobial resistance, while underscoring the need for continued research investment, regulatory innovation, and healthcare system adaptation to translate these promising strategies into clinical practice.

## 5. The One Health Framework for Integrated AMR Surveillance and Control

Antimicrobial resistance transcends traditional disciplinary boundaries, operating at the intersection of human, animal, and environmental health in ways that defy conventional sectoral approaches. Resistant microorganisms, resistance genes, and mobile genetic elements circulate continuously between humans, animals, food production systems, wildlife, and the environment, creating a highly interconnected network that cannot be understood, or effectively controlled, through isolated interventions [32]. This recognition has progressively established the One Health framework as the conceptual foundation of modern AMR prevention, emphasising that human health is inseparable from animal health and environmental integrity [25,38,40].

Initially developed to strengthen collaboration between human and veterinary medicine, One Health has evolved into a comprehensive systems-based approach that integrates microbiology, ecology, environmental sciences, public health, agriculture, data science, and health policy [41]. Within this framework, antimicrobial resistance is no longer viewed as the consequence of antibiotic exposure within individual patients but as the outcome of complex biological and societal interactions occurring across multiple ecosystems [40]. This paradigmatic shift has profound implications for surveillance strategies, intervention design, and policy development, requiring coordination across sectors that have historically operated in isolation.

The One Health approach recognises that the health of humans, domestic and wild animals, plants, and the wider environment are closely linked and interdependent. In the context of AMR, this interdependence manifests through multiple pathways: the use of antibiotics in agriculture selects for resistant bacteria that can be transmitted to humans through the food chain; antimicrobial residues and resistant organisms from healthcare settings and farms enter the environment through wastewater and runoff; and environmental bacteria serve as reservoirs of resistance genes that can be mobilised into human pathogens through horizontal gene transfer [38,40]. Understanding and interrupting these transmission pathways requires integrated surveillance, collaborative research, and coordinated policy action across all sectors.

### 5.1. The Human-Animal-Environmental Interface: A Continuous Resistome

Resistance does not emerge within a single sector but rather across a continuum of interconnected ecological compartments. Antibiotics administered to humans, livestock, aquaculture, and companion animals exert selective pressure across diverse ecological niches, favouring the survival and proliferation of resistant bacteria wherever antimicrobial residues are present [38]. Resistant bacteria and antimicrobial residues subsequently enter wastewater, agricultural soils, rivers, and food production systems, where they interact with environmental microorganisms through horizontal gene transfer, creating an expanding environmental resistome that serves as a reservoir for resistance genes that can be mobilised into clinical settings [39,40].

Whole-genome sequencing has demonstrated that identical resistance determinants, including carbapenemases, extended-spectrum β-lactamases, and plasmid-mediated colistin resistance genes, can be identified across clinical isolates, food-producing animals, environmental samples, and wildlife [32]. These observations indicate that resistance genes frequently move between ecological compartments rather than remaining confined to individual bacterial populations [34]. The mobility of these genetic elements, often carried on transmissible plasmids and other mobile genetic elements, enables rapid dissemination across species boundaries and geographical regions, complicating efforts to trace and contain resistance emergence [40].

The food chain represents a particularly important interface within this network. Modern food production systems facilitate interactions between antimicrobial use in agriculture, bacterial evolution, environmental contamination, and human exposure [39]. Intensive farming practices, particularly in livestock and aquaculture, involve substantial antimicrobial use for disease prevention and growth promotion, creating selective pressure that favours the emergence of resistant bacteria in animal populations. These bacteria can be transmitted to humans through direct contact, consumption of contaminated food products, or environmental pathways, including contamination of water sources and agricultural soils [39]. Recent evidence suggests that addressing antimicrobial resistance within food systems requires coordinated surveillance extending from primary production to food processing and retail distribution, reinforcing the need for integrated policies that encompass both public health and agricultural practices [39].

The role of wastewater treatment plants, surface waters, sediments and agricultural runoff as hotspots for resistance gene exchange has been described in Section 2.2, and their use as surveillance substrates in Section 3.4; that material is not repeated here. What is specific to the present section is the direction of flow between compartments. These environments bring together bacterial populations of human, veterinary and industrial origin at high density, in the presence of sub-inhibitory antimicrobial concentrations, and thereby function less as endpoints than as exchange interfaces from which determinants can re-enter clinical settings [14,38,40]. The implication for surveillance design is that sampling strategies must be able to attribute a detected determinant to a compartment of origin, which is a substantially harder requirement than detection alone.

### 5.2. AI-Enabled Surveillance Integration and Predictive Intelligence

One of the greatest strengths of the One Health approach lies in its ability to integrate information generated across different sectors, creating a more complete picture of resistance emergence and transmission [32]. Historically, surveillance systems have operated independently, with separate monitoring programmes for hospitals, veterinary medicine, food production, and environmental microbiology. Although valuable in their own right, these fragmented systems often fail to identify the broader transmission pathways through which antimicrobial resistance emerges and spreads, limiting opportunities for early intervention and coordinated response [36].

Recent advances in genomics, digital epidemiology, and artificial intelligence are beginning to overcome these limitations. Whole-genome sequencing allows bacterial isolates collected from different ecological compartments to be compared with unprecedented precision, reconstructing transmission pathways that would previously have remained undetected [32]. By generating high-resolution phylogenetic trees and tracking the movement of mobile genetic elements, genomic surveillance can identify connections between human, animal, and environmental reservoirs, revealing transmission networks that span sectors and geographical boundaries [33,34]. These capabilities have been particularly valuable for understanding the spread of carbapenem-resistant Enterobacterales, extended-spectrum β-lactamase-producing organisms, and colistin-resistant bacteria, where genomic data have revealed previously unrecognised links between clinical and environmental sources [36].

The exponential growth of available surveillance data has created new analytical challenges that demand innovative solutions. Modern One Health programmes generate vast quantities of information from hospitals, diagnostic laboratories, veterinary services, food production facilities, wastewater monitoring networks, satellite observations, climate databases, and genomic repositories [32]. Integrating these heterogeneous datasets, which vary in format, resolution, quality, and temporal and spatial scales, exceeds the capabilities of conventional analytical methods, creating a critical bottleneck in the translation of surveillance data into actionable intelligence [30].

Artificial intelligence increasingly functions as the connective infrastructure linking these diverse information sources, enabling the integration and analysis of data that would otherwise remain siloed [28]. Machine learning algorithms can identify correlations among environmental variables, antimicrobial consumption patterns, bacterial genomics, climate indicators, animal health records, and human disease surveillance, revealing transmission dynamics that may otherwise remain undetected [20]. These analytical capabilities are particularly valuable for uncovering complex, non-linear relationships that characterise AMR transmission across sectors, where multiple factors interact in ways that are difficult to capture using traditional statistical approaches [30].

Beyond descriptive analyses, AI enables the development of dynamic risk models capable of continuously updating predictions as new information becomes available [26]. Rather than merely documenting resistance after it has occurred, integrated One Health surveillance increasingly seeks to anticipate where resistant pathogens are most likely to appear and how they may subsequently spread [62]. These predictive capabilities are essential for proactive intervention, enabling public health authorities to implement targeted measures before widespread dissemination occurs [30].

As discussed in Section 2.2 and Section 3.4, environmental surveillance through wastewater monitoring, agricultural runoff analysis, and wildlife screening represents a critical complement to clinical surveillance. The operational implementation of these approaches requires harmonised protocols, sustainable funding mechanisms, and integration with national and international reporting systems to translate environmental data into actionable public health intelligence; the specific barriers encountered by existing implementations are set out in Section 3.7.

Importantly, AI does not replace microbiological expertise or epidemiological investigation. Rather, it enhances the ability of multidisciplinary teams to interpret increasingly complex datasets and to transform fragmented information into actionable public health knowledge [28]. The effective use of AI in One Health surveillance requires close collaboration between data scientists, microbiologists, epidemiologists, veterinarians, environmental scientists, and public health practitioners, ensuring that analytical outputs are appropriately interpreted and applied in context.

This evolution reflects a broader conceptual shift from surveillance as a passive reporting activity, documenting resistance after it has emerged, toward surveillance as an active early, warning system capable of generating actionable intelligence for prevention and control. The transition from retrospective to prospective surveillance represents a fundamental transformation in how AMR threats are identified and managed, with the potential to substantially reduce the burden of resistant infections through earlier and more targeted interventions.

### 5.3. Governance, Policy and International Collaboration for One Health Implementation

Technological innovation alone will not halt the global expansion of antimicrobial resistance. Effective implementation of One Health strategies requires coordinated governance capable of integrating healthcare systems, veterinary services, environmental agencies, food safety authorities, academic institutions, and international organisations [26]. The complexity of AMR transmission, spanning multiple sectors, disciplines, and geographical boundaries, demands governance structures that transcend traditional institutional silos and enable coordinated action across the full spectrum of relevant stakeholders.

Recent international initiatives have increasingly recognised this need. The WHO Global Research Agenda for AMR has identified integrated surveillance, equitable access to diagnostics, genomic monitoring, implementation science, and digital innovation among the highest global research priorities [26]. Similarly, the EPI-Net One Health reporting guidelines have proposed standardised approaches for collecting and reporting antimicrobial consumption and resistance data across human, animal, and environmental sectors, facilitating international comparisons and collaborative research [43]. These initiatives represent important steps toward harmonised surveillance and coordinated action, but substantial work remains to translate these frameworks into effective practice at national and regional levels.

The updated WHO Global Action Plan on Antimicrobial Resistance 2026–2036 was adopted by the World Health Assembly in May 2026 [102]. The same plan was subsequently adopted by the World Organisation for Animal Health, which gives it standing across the animal-health sector as well as the human one [103]. This dual adoption is not a procedural detail. The Quadripartite architecture (FAO, UNEP, WHO and WOAH) has often been criticised for producing parallel rather than joint commitments, and the endorsement of a single action plan by both the human and animal health bodies establishes a shared set of targets against which the integrated surveillance described in this section can actually be assessed. The remaining asymmetry concerns the environmental pillar, where UNEP’s role is well articulated conceptually but is supported by weaker reporting obligations than those in place for the human and animal sectors.

Nevertheless, considerable disparities remain between high-income and resource-limited settings. Many countries continue to lack robust laboratory infrastructure, interoperable surveillance systems, or sustainable genomic capacity [23]. These inequalities risk widening the global AMR gap, particularly as advanced technologies become increasingly central to resistance monitoring and response. The digital divide, the unequal access to computational resources, technical expertise, and data infrastructure, threatens to concentrate the benefits of technological innovation in high-income settings while leaving low- and middle-income countries without the tools needed to effectively monitor and respond to AMR threats [23].

The infrastructure barriers facing low- and middle-income countries are not limited to laboratory capacity. They include: unreliable electricity and internet connectivity, which prevent the deployment of cloud-based bioinformatic pipelines; shortage of trained bioinformaticians and data scientists, which limits the ability to interpret genomic or AI-generated outputs; absence of national data governance frameworks, which prevents legal cross-border data sharing; and dependence on external funding for equipment and reagents, which undermines the sustainability of surveillance programmes. Addressing these barriers requires not only technology transfer but also sustained investment in workforce development, national data infrastructure, and regional reference laboratory networks. Without such investment, the technologies described in this review will remain concentrated in a small number of well-resourced institutions, and the global AMR gap will continue to widen.

Future policy development should therefore prioritise not only technological innovation but also equitable implementation. Investments in laboratory networks, workforce training, data-sharing platforms, and regulatory harmonisation will be essential to ensure that the benefits of digital surveillance and artificial intelligence are accessible across diverse healthcare settings rather than remaining concentrated within a limited number of highly resourced institutions [43]. International cooperation, technology transfer, and capacity-building initiatives can help bridge the global AMR gap, promoting equitable access to advanced surveillance and diagnostic tools while strengthening local expertise and infrastructure.

The One Health framework is undergoing a profound transformation. Rather than serving solely as a conceptual model for interdisciplinary collaboration, it is progressively evolving into a predictive ecosystem in which genomics, artificial intelligence, environmental monitoring, and digital surveillance operate as interconnected components of a single global infrastructure [32,62]. This evolution reflects a broader recognition that effective AMR control requires not only understanding current resistance patterns but also anticipating future threats and enabling proactive intervention.

Within this emerging paradigm, hospitals become only one node of a much broader surveillance network. Wastewater monitoring may provide early signals of resistance emergence at community level; veterinary surveillance may identify novel resistance mechanisms before their appearance in humans; climate data may help anticipate ecological conditions favouring bacterial dissemination; and artificial intelligence may integrate these diverse signals into real-time risk assessments capable of supporting both clinical and public health decision-making [62]. The convergence of these data streams creates a comprehensive, continuously updated picture of AMR dynamics that can inform coordinated action across sectors and scales.

The integration of One Health principles with advanced technologies also enables new approaches to intervention design and evaluation. Predictive models can identify potential intervention points in transmission networks, enabling targeted measures that interrupt spread while minimising unintended consequences. Real-time surveillance can monitor the effectiveness of interventions, enabling adaptive management and continuous improvement. The feedback loops between surveillance, prediction, and intervention create a learning system that becomes increasingly effective over time, as experience and data inform model refinement and strategy adjustment.

Ultimately, the future success of antimicrobial resistance control will depend less on isolated technological breakthroughs than on the ability to connect these innovations within an integrated global framework [41]. One Health therefore represents not simply an interdisciplinary philosophy but the operational architecture through which next-generation AMR preparedness can be achieved. The challenge lies in translating this vision into practice, building the institutional capacity, political will, and international cooperation needed to implement One Health surveillance and intervention at scale.

The integration of One Health principles with the technological advances discussed in the previous sections, particularly AI, genomics, and digital surveillance, offers a powerful foundation for addressing the complex, multi-sectoral challenge of antimicrobial resistance. The Digital One Health Intelligence Cycle proposed in Figure 2 illustrates how these components can be linked in a continuous learning system that supports proactive AMR preparedness across human, animal, and environmental sectors. By connecting surveillance, prediction, and intervention through intelligent data integration, it becomes possible to move from reactive response toward proactive prevention, anticipating and mitigating resistance threats before they become established.

## 6. Discussion

The evolving landscape of antimicrobial resistance demands a fundamental rethinking of current strategies. That rethinking must move beyond traditional disciplinary boundaries toward an integrated, systems-based approach. As this review has shown, AMR can no longer be understood as a purely clinical or microbiological problem. It has become a paradigmatic example of a global polycrisis. Pandemics, climate change, armed conflicts, population mobility and health inequalities interact synergistically to accelerate the emergence and dissemination of resistant pathogens. Each of these interactions has distinct implications for prevention, surveillance and therapeutic innovation.

Traditional antimicrobial stewardship programmes remain essential, but they have proven insufficient to curb the global expansion of resistance. The COVID-19 pandemic illustrated this vividly. Stewardship gains were reversed within months once healthcare systems were overwhelmed, diagnostic capacity was limited, and empirical prescribing became the default clinical strategy. A similar pattern is visible in conflict zones and in resource-limited settings, where broad-spectrum use is driven by necessity rather than by clinical preference. These examples point to structural determinants that behavioural interventions alone cannot reach.

The limitations of stewardship-focused approaches reveal a critical gap in current AMR strategies. Such approaches address the consequences of resistance rather than its causes. They do not engage with the environmental reservoirs of resistance genes, with the ecological conditions that facilitate horizontal gene transfer, or with the socioeconomic factors that drive inappropriate prescribing. A more comprehensive approach is therefore required, one that extends beyond healthcare facilities to the broader ecosystems in which resistance emerges and spreads.

The integration of artificial intelligence, genomics and digital surveillance offers real opportunities to address these limitations. Predictive models can identify emerging resistance patterns before they become clinically apparent. Whole-genome sequencing enables reconstruction of transmission networks with considerable precision. Metagenomic and wastewater surveillance can provide early warning at population and ecosystem levels.

Several constraints, however, stand between these capabilities and their realisation. AI models depend on high-quality, representative training data, and algorithms developed in high-income settings often perform less well when applied to geographically distinct populations. The limited interpretability of certain deep learning models remains a barrier to clinical acceptance. Implementation requires substantial computational infrastructure, technical expertise and sustained financing. As set out in Section 3.7, existing deployments are overwhelmingly sector-specific, retrospective and project-funded, and no fully integrated One Health platform is currently in routine operation. There is a genuine risk that technologies designed to combat AMR will widen rather than narrow the global health gap.

Three claims that appear frequently in the AMR literature are not, on the current evidence, sustainable. First, genomic prediction of resistance cannot yet replace phenotypic susceptibility testing for organisms whose resistance is polygenic or expression-dependent, and should be used alongside phenotypic methods rather than instead of them [34]. Second, wastewater surveillance cannot yet be converted into population prevalence estimates without agreed denominators and sampling strategies; what it provides is early warning, not quantification [51]. Third, AI-designed antibacterial candidates have not yet demonstrated clinical efficacy and safety in humans, and their contribution remains preclinical [30,74]. Recognising these limits is not a reason to slow investment but a precondition for designing studies that can actually establish benefit.

Several specific research questions follow directly from the gaps identified in this review. First, how do AMR prediction models perform under prospective external validation in the settings where they would be deployed, and what reporting standards should govern their clinical evaluation? Second, what denominators and sampling frameworks can make environmental surveillance quantitatively interpretable rather than only qualitatively indicative? Third, which phage products can demonstrate efficacy in randomised controlled trials for biofilm-associated infections, and what regulatory pathways can accommodate their personalised nature? Fourth, can CRISPR-based antimicrobials achieve sufficient in vivo delivery efficiency to enter clinical trials? Fifth, what is the health-economic case for AI-assisted stewardship in resource-limited settings, where the marginal value of improved prescribing may be highest but the infrastructure constraints are most severe? Sixth, what governance and data-sharing arrangements can make integrated One Health surveillance operational without reproducing existing inequities in data ownership and analytical capacity? These questions define a research agenda that is both scientifically tractable and directly relevant to the policy decisions now being taken.

The growing therapeutic arsenal beyond conventional antibiotics represents a welcome diversification. Most of these strategies, however, remain at an early stage of development. Bacteriophage therapy requires careful matching of phages to individual bacterial isolates. CRISPR-based antimicrobials face persistent difficulties in efficient in vivo delivery, as the partial reductions achieved in recent murine work illustrate [88]. Antimicrobial peptides must overcome toxicity, proteolytic instability and production cost before systemic use becomes feasible, although recent peptides that potentiate β-lactams rather than acting alone suggest that combination strategies may lower the doses at which these constraints bind [91].

Nanomedicine and related platform technologies face comparable hurdles in safety, manufacturing complexity and clinical validation. It is worth noting that the same technological direction, the combination of drug repurposing with nanotherapeutic delivery, is being pursued in parallel against fungal pathogens [104]. This parallel development suggests that these are cross-cutting platform strategies rather than expedients specific to bacterial resistance, and that regulatory and manufacturing lessons are likely to transfer between the two fields.

It is unlikely that any single emerging therapy will replace antibiotics. The future of antimicrobial treatment will more plausibly involve the rational integration of conventional agents with innovative platforms, tailored to individual pathogens, resistance mechanisms and host characteristics. Artificial intelligence may assist in selecting optimal combinations. Genomics may identify actionable resistance determinants. Emerging therapies may expand the options available for previously untreatable infections. The path from laboratory discovery to clinical implementation nonetheless remains long, and the regulatory, economic and logistical obstacles require coordinated action across academia, industry and government.

Perhaps the most important insight emerging from this review is that AMR cannot be addressed within healthcare settings alone. Resistant bacteria, resistance genes and mobile genetic elements circulate continuously between humans, animals, food production systems and the environment. A coordinated, multisectoral approach is therefore unavoidable. The One Health framework provides the conceptual and operational architecture for such integration, linking human and veterinary medicine with environmental surveillance, food safety and agricultural practice.

The integration of advanced technologies within this framework is a particularly promising development. Artificial intelligence can serve as connective infrastructure, linking clinical, veterinary, environmental, genomic and climatic data streams into shared analytical platforms. Predictive models can identify emerging hotspots, forecast outbreak risk and inform coordinated prevention. The translation of One Health principles into operational practice nonetheless remains difficult. It requires governance structures, funding mechanisms and accountability frameworks that transcend institutional silos, and these are considerably harder to build than the technical infrastructure itself [105,106]. Stewardship implementation in low- and middle-income settings has illustrated the same point: even well-designed toolkits encounter structural barriers when laboratory capacity, workforce and financing are constrained [107,108].

Despite these advances, the global distribution of AMR surveillance and therapeutic capacity remains substantially unequal. Low- and middle-income countries bear the greatest burden of resistant infections, yet frequently lack the laboratory infrastructure, trained personnel and financial resources required for advanced technologies [23,24]. The resulting diagnostic gap perpetuates empirical prescribing and forecloses opportunities for targeted therapy. Addressing these inequities must become a central priority of global AMR strategy. This will require investment in laboratory networks, workforce training, data-sharing platforms and quality management systems, supported by international partnership and technology transfer. Without such investment, the benefits of innovation will remain concentrated in a small number of well-resourced institutions.

Several priority areas for coordinated action follow from this analysis. Surveillance systems should be strengthened through interoperable data models, harmonised reporting standards and agreed minimum reporting sets, so that clinical, veterinary and environmental data can be analysed jointly rather than in parallel [32,63]. Stewardship should be supported by electronic prescribing, audit-and-feedback and AI-assisted decision support, with the same governance and implementation constraints acknowledged as for any other health-system intervention [30,35,107]. Innovation in phage therapy, CRISPR-based antimicrobials, antimicrobial peptides, nanomedicine and microbiota-directed therapies must be supported by trial-stage evidence, standardised manufacturing and adaptive regulatory pathways [79,85,89,94]. Prevention through vaccination, water and sanitation infrastructure, and infection prevention and control should be funded on the same basis as therapeutic innovation [54,101,102]. Equity requires technology transfer, infrastructure investment and affordable access in the settings carrying the greatest burden [23,24,80]. Operational One Health requires governance structures, funding and cross-sector accountability that currently exist only in a minority of countries [43,105,106]. AI integration requires predictive modelling, real-time surveillance and interoperable platforms, and remains prospective rather than deployed [28,30,45,55,62].

Effective AMR control will finally require strengthened international governance. The WHO GLASS programme has demonstrated the feasibility of coordinated surveillance, but the integration of genomic, environmental and veterinary data into common platforms remains incomplete. Standardised protocols, interoperable systems and harmonised reporting guidelines are prerequisites for meaningful international comparison. The political and economic drivers of AMR must also be addressed directly. These include agricultural antibiotic use, pharmaceutical manufacturing practice and healthcare financing, and they require policy frameworks that promote sustainable use across all sectors while supporting equitable access to diagnostics and novel therapeutics.

Several limitations of this review should be acknowledged. This is a narrative rather than a systematic review. The literature was selected purposively to illustrate the interaction between global drivers, emerging technologies and integrated responses, and no formal risk-of-bias appraisal or quantitative synthesis was undertaken. The restriction to English-language sources, combined with the well-documented over-representation of high-income settings in AMR surveillance and genomics literature, means that the evidence summarised here under-represents precisely those regions carrying the greatest burden of resistant infections [23]. Several of the technologies discussed rest largely on preclinical or early-phase evidence, including CRISPR-based antimicrobials, nanomedicine, host-directed therapies and AI-designed antibacterials. Readers should not infer a degree of translational readiness that the current data do not support. The fields covered are also moving quickly, and the policy landscape changed substantially between 2024 and 2026; specific figures, targets and pipeline data should be checked against the most recent WHO and Quadripartite reporting at the time of reading. Finally, the breadth of the topics covered means that no single area could be treated with the depth of a dedicated systematic review, and readers seeking exhaustive coverage of any subtopic are referred to the specialised literature cited throughout.

Ultimately, translating these priorities into practice will require sustained political commitment, adequate funding and international cooperation. It will also require a shift in how antimicrobial resistance is understood: not as a technical problem with a technical solution, but as a societal challenge that demands collective action across sectors.

## 7. Conclusions

Antimicrobial resistance has become one of the defining public health challenges of the twenty-first century. It is no longer driven solely by inappropriate antibiotic use; it now reflects the interaction of pandemics, climate change, armed conflict, global mobility, environmental disruption and persistent health inequalities. At the same time, technological advances are reshaping the response. Artificial intelligence, genomics, metagenomics, digital surveillance and predictive analytics enable earlier detection, more accurate diagnosis, improved stewardship and accelerated drug discovery. Parallel developments in phage therapy, CRISPR-based technologies, antimicrobial peptides, microbiota-directed interventions and nanomedicine are expanding the therapeutic landscape beyond conventional antibiotics. The greatest opportunity lies not in any single innovation, but in their integration. The convergence of these technologies within a One Health framework makes it possible to move from a reactive model, centred on treating resistant infections, toward a proactive system capable of anticipating and mitigating resistance across human, animal and environmental ecosystems. This transition should not be presented as inevitable. Current implementations remain partial, sector-specific and dependent on project funding. Realising the potential described here requires political will, institutional capacity and a commitment to equity that ensures the benefits of innovation are widely shared. The future of AMR preparedness will depend on both continued antimicrobial discovery and the construction of intelligent, interconnected and equitable systems. Neither is sufficient alone: without new agents, the therapeutic arsenal will continue to narrow, and without integrated surveillance, prevention and stewardship systems, new agents will be deployed under the same selective pressures that have driven the current crisis. The two priorities are complementary rather than competing, and both require sustained political will, institutional capacity and a commitment to equity.

## Figures and Tables

**Table 1:** Summary of surveillance, diagnostic and therapeutic technologies for AMR: application, current stage, quantitative performance, turnaround time, cost and infrastructure needs, and translational maturity. Abbreviations: AMR, antimicrobial resistance; AI, artificial intelligence; AST, antimicrobial susceptibility testing; CRISPR, clustered regularly interspaced short palindromic repeats; MALDI-TOF, matrix-assisted laser desorption/ionization time-of-flight; ML, machine learning; PCR, polymerase chain reaction; WGS, whole-genome sequencing. Values of sensitivity and specificity are indicative and are drawn from the cited primary literature; where formal diagnostic accuracy studies are lacking, qualitative descriptors are used.

Technology	Application	Current Stage	Sensitivity/ Specificity	Turnaround Time	Cost/ Infrastructure Needs	Translational Maturity
*Surveillance technologies *						
Genomic surveillance (WGS)	Transmission tracking; resistance-gene detection; outbreak reconstruction	Routine use in many high-income settings; expanding in LMICs	High (>95% for well-characterised determinants; lower for polygenic resistance)	24–72 h	High (sequencer, bioinformatic pipeline, trained personnel)	Established in routine use
Metagenomics (culture-independent)	Direct pathogen and resistome detection from clinical and environmental samples	Routine use in reference laboratories; expanding to clinical workflows	High for pathogen detection; variable for resistance-gene attribution	24–48 h	High (sequencer, curated databases, bioinformatic expertise)	Established in reference laboratories
Wastewater monitoring	Population-level early warning; environmental resistome profiling	National pilot programmes; repurposed SARS-CoV-2 networks	Qualitative (no agreed denominator; cannot be converted to prevalence)	24–48 h	Low per sample; requires network infrastructure and sampling strategy	In evaluation; partial national deployment
Digital surveillance (EHR, mobility, climate data)	Real-time situational awareness; predictive epidemiology	Sector-specific implementations; no cross-sector platform	Not applicable (aggregate signals, not individual diagnosis)	Near real-time to 24 h	Moderate to high (data infrastructure, governance, interoperability)	In evaluation; sector-specific
Integrated One Health AI platform	Cross-sector predictive intelligence; coordinated preparedness	No operational system to our knowledge	Not applicable (no deployed system)	Not applicable	Very high (semantic, legal, methodological and financial barriers)	Preclinical/ conceptual
*Diagnostic technologies*						
MALDI-TOF with ML	Rapid susceptibility prediction	Clinical evaluation in selected centres	80–95% (centre-dependent)	1–4 h from positive culture	Moderate (MALDI-TOF instrument; ML software)	In clinical evaluation
Syndromic multiplex PCR panels	Pathogen and resistance-marker identification from positive cultures	Routine use in many hospitals	90–99% for targeted pathogens	1–2 h	Moderate (PCR platform; cartridge costs)	Established in routine use
Direct-from-blood-culture rapid AST	Phenotypic susceptibility results hours earlier than conventional workflows	Clinical evaluation	85–95% concordance with conventional AST	4–8 h	Moderate to high (platform-specific)	In clinical evaluation
Isothermal amplification (LAMP, RPA)	Point-of-care detection of resistance genes	Laboratory and field evaluation	85–95%	15–60 min	Low (instrument-light; field-deployable)	In evaluation
CRISPR-based diagnostics	Point-of-care resistance-gene detection	Early clinical evaluation	90–99% (target-dependent)	30–60 min	Low to moderate (in development)	Preclinical/early clinical
Host-response markers (procalcitonin, transcriptomic signatures)	Reducing unnecessary antibiotic initiation	Clinical evaluation and routine use in some settings	Variable (70–90% for discriminating bacterial from viral infection)	1–4 h	Low to moderate	In clinical evaluation; partial routine use
*Therapeutic technologies*						
Bacteriophage therapy	MDR infections; biofilm-associated infections	Compassionate use; early-phase trials	Not applicable (pathogen-specific; requires matching)	Days to weeks (matching required)	Moderate (manufacturing; regulatory pathway)	In clinical evaluation
CRISPR-based antimicrobials	Targeted elimination of resistance determinants or pathogens	Preclinical	Not applicable (preclinical)	Not applicable	High (delivery, manufacturing, regulatory)	Preclinical/proof-of-concept
Antimicrobial peptides	MDR infections; topical and systemic applications	Early clinical and preclinical development	Variable; broad-spectrum in vitro	Not applicable (systemic administration)	High (production cost; stability)	Early clinical; preclinical
Nanomedicine	Targeted antimicrobial delivery; biofilm penetration	Experimental/ preclinical	Not applicable (delivery platform, not diagnostic)	Not applicable	High (manufacturing complexity)	Preclinical
Microbiota-directed therapies	Restoration of colonisation resistance; MDR decolonisation	Clinical use in selected indications	Variable; heterogeneous clinical evidence	Days to weeks	Moderate	Clinical use in selected indications
Host-directed therapies	Adjunctive immunotherapy in severe infections	Early clinical evaluation	Not applicable (host-directed)	Not applicable	Moderate to high	Early clinical
Photodynamic therapy	Localised infections; biofilm-associated diseases	Clinical use in selected localised infections	Effective against MDR pathogens in vitro and in localised infections	Minutes to hours (per session)	Low to moderate (light delivery systems)	Clinical use in selected indications
Vaccination	Prevention of infections that drive AMR	Established clinical and public health use	Varies by vaccine (60–95%)	Weeks to years (immunity onset)	Moderate (programmatic delivery)	Established in routine use

**Table 2:** Emerging non-antibiotic therapeutic strategies for antimicrobial resistance: mechanisms, advantages, limitations, and current stage of development. Abbreviations: AMR, antimicrobial resistance; CRISPR, clustered regularly interspaced short palindromic repeats; AMPs, antimicrobial peptides; PDT, photodynamic therapy; FMT, faecal microbiota transplantation; WASH, water, sanitation and hygiene.

Strategy	Mechanism of Action	Main Advantages	Current Limitations	Development Stage	Primary One Health Compartment(s) of Application	Key References
Bacteriophage therapy	Host-specific bacteriophages selectively infect and lyse bacterial pathogens	High specificity; activity against biofilm-associated infections; efficacy against multidrug-resistant bacteria	Narrow host range; regulatory challenges; potential bacterial resistance to phages	Compassionate use and early clinical trials	Human (compassionate use, early trials); food production (authorised anti-*Listeria* products for ready-to-eat foods and food-contact surfaces); aquaculture and livestock	[79,82,83,84]
CRISPR-based antimicrobials	Sequence-specific targeting of bacterial genomes or resistance genes using CRISPR-Cas systems	Precision therapy; preservation of commensal microbiota; potential elimination of resistance determinants	Efficient in vivo delivery remains challenging; predominantly preclinical evidence	Preclinical	Human (preclinical); veterinary and food-producing animals (preclinical, targeted decolonisation)	[85,86,87,88]
Antimicrobial peptides (AMPs)	Disruption of bacterial membranes combined with immunomodulatory activity	Broad-spectrum antimicrobial activity; reduced propensity for resistance; anti-biofilm effects	Toxicity; proteolytic instability; high production costs	Early clinical and preclinical development	Human (topical use; systemic in development); livestock (feed additives as alternatives to growth promoters)	[89,90,91]
Nanomedicine	Nanoparticles act as antimicrobial agents or targeted drug-delivery systems	Enhanced drug delivery; improved biofilm penetration; reduced systemic toxicity	Safety concerns; manufacturing complexity; limited clinical validation	Experimental/ Preclinical	Human (experimental); environment (water treatment, surface disinfection); food (packaging and processing surfaces)	[92,93]
Microbiota-directed therapies	Restoration of intestinal colonisation resistance through microbiome modulation	Reduction of intestinal multidrug-resistant bacterial carriage; restoration of microbial diversity	Heterogeneous clinical evidence; standardisation and regulatory issues	Clinical use in selected indications	Human (selected indications); veterinary (competitive exclusion products in poultry, established use)	[94,95,96]
Host-directed therapies	Enhancement or modulation of host immune responses rather than direct bacterial killing	Reduced selective pressure for resistance; adjunctive effect with antibiotics	Limited pathogen-specific evidence; few approved indications	Early clinical evaluation	Human only (early clinical evaluation)	[97,98]
Photodynamic therapy	Light-activated photosensitisers generate reactive oxygen species causing microbial cell damage	Low likelihood of resistance development; effective against biofilms and localised infections	Suitable mainly for superficial or localised infections; limited systemic application	Clinical use in selected localised infections and ongoing experimental development	Human (dentistry, dermatology, chronic wounds); food processing (surface decontamination); aquaculture (experimental)	[99,100]
Vaccination (Section 4.8)	Induction of protective immunity reducing infection incidence and antibiotic use	Largest existing evidence base for reducing antimicrobial consumption and resistance	Serotype replacement; coverage and access barriers	Established clinical and public health use	Human, companion animals and food-producing animals simultaneously; the only strategy with large-scale established use in all three	[101]

## Data Availability

No new data were created or analyzed in this study. Data sharing is not applicable to this article.
